# Supplementary material for: PHF3 regulates neuronal gene expression through the Pol II CTD reader domain SPOC
Source: Nat Commun. 2021 Oct 19;12:6078. doi: 10.1038/s41467-021-26360-2 (PMC8526623; doi:10.1038/s41467-021-26360-2)
Supplement: Supplementary file 1 — Supplementary Information [file 41467_2021_26360_MOESM1_ESM.pdf]

SUPPLEMENTARY TABLES

Supplementary Table 1. CTD peptides

	Sequence	Experiment	Source
CTD unmodified	FAM-PSYSPTSPSYSPTSPS	Fluorescence anisotropy	Clonestar
CTD 2xpS2	FAM-PSYpSPTSPSYpSPTSPS	Fluorescence anisotropy	Clonestar
CTD 2xpS2pS5	FAM-PSYpSPTpSPSYpSPTpSPS	Fluorescence anisotropy	Clonestar
CTD 2xpS2pS7	FAM-PSYpSPTSPpSYpSPTSPpS	Fluorescence anisotropy	Clonestar
CTD 2xpS5	FAM-PSYSPTpSPSYSPTpSPS	Fluorescence anisotropy	Clonestar
CTD 2xpS7	FAM-PSYSPTSPpSYSPTSPpS	Fluorescence anisotropy	Clonestar
CTD 2xpS5pS7	FAM-PSYSPTpSPpSYSPTpSPpS	Fluorescence anisotropy	Clonestar
CTD pS2	FAM-PSYpSPTSPSYSPTSPS	Fluorescence anisotropy	Clonestar
CTD 2xpS2	Atto488-PSYpSPTSPSYpSPTSPS	Fluorescence correlation spectroscopy	Clonestar
CTD 2xpS2pS5	Atto488-PSYpSPTpSPSYpSPTpSPS	Fluorescence correlation spectroscopy	Clonestar
CTD unmodified	PSYSPTSPSYSPTSPS	X-ray crystallography	Clonestar
CTD 2xpS2	PSYpSPTSPSYpSPTSPS	X-ray crystallography	Clonestar
CTD 2xpS2pS5	PSYpSPTpSPSYpSPTpSPS	X-ray crystallography	Clonestar
CTD 2xpS2pS7	PSYpSPTSPpSYpSPTSPpS	X-ray crystallography	Clonestar

Supplementary Table 2. X-ray data collection and refinement

	**SMet apoSPOC**	**SPOC:2xpS2pS5**	**SPOC:2xpS2**	**SPOC:2xpS2pS7**
Source ESRF	ID29		MASSIF ID30a1	
Wavelength (Å)	0.979	1.072	0.966	0.966
Resolution (Å)	48.78-2.59 (2.71-2.59)	44.5-2.85 (3.0-2.85)	47.47-1.93 (1.97-1.93)	49.1-1.75 (1.78-1.75)
Space group	$P2_1$	$P3_2$	$P2_12_12_1$	$P2_12_12_1$
Unit cell (Å, º)	69.75, 69.31, 127.22; β=100.09	67.83, 67.83, 178.11	54.07, 68.02, 99.17	56.22, 68.11, 100.87
Molecules (a.u.)	8	4	2	2
Unique reflections	32812 (2252)	21184 (3104)	27050 (1262)	39627 (1968)
Completeness (%)	87.8 (50.3)	98.8 (99.5)	96.0 (69.0)	99.2 (91.3)
R_{merge}[b]	0.105 (0.867)	0.157 (1.432)	0.076 (1.525)	0.058 (2.753)
R_{meas}[c]	0.126 (1.178)	0.183 (1.662)	0.083 (1.69)	0.065 (3.143)
CC(1/2)	0.997 (0.520)	0.993 (0.285)	0.990 (0.326)	0.990 (0.230)
Multiplicity	6.0 (3.6)	3.5 (3.6)	6.2 (5.1)	4.4 (4.1)
I/sig(I)	10.5 (1.1)	6.6 (0.9)	11.4 (1.0)	11.8 (0.5)
B_{Wilson} (Å^2)	51.7	52.9	41.3	35.1
R_{work}[e]/R_{free}[f] (%)	20.6/25.9	19.2/22.7	18.4/22.6	19.5/23.2
r.m.s.d. bonds (Å)	0.003	0.005	0.007	0.015
r.m.s.d. angles (°)	0.605	1.385	0.949	1.329

[a] Values in parentheses are for the highest resolution shell.

[b] $$R_{merge} = \frac{\sum_{hkl}\sum_{i=1}^{N}\left|I_{i(hkl)} - \bar{I}_{(hkl)}\right|}{\sum_{hkl}\sum_{i=1}^{N} I_{i(hkl)}}$$

[c] $$R_{meas} = \frac{\sum_{hkl}\sqrt{N/(N-1)}\sum_{i=1}^{N}\left|I_{i(hkl)} - \bar{I}_{(hkl)}\right|}{\sum_{hkl}\sum_{i=1}^{N} I_{i(hkl)}}$$

where $\bar{I}_{(hkl)}$ is the mean intensity of multiple $I_{i(hkl)}$ observations of the symmetry-related reflections, N is the redundancy

[e] $$R_{work} = \frac{\sum\left||F_{obs}| - |F_{calc}|\right|}{\sum|F_{obs}|}$$

[f] R_{free} is the cross-validation R_{factor} computed for the randomly chosen test set of reflections (5 %) which are omitted in the refinement process.

Supplementary Table 3. Antibodies

	SOURCE	IDENTIFIER
Mouse anti-FLAG M2-peroxidase	Sigma	A8592; 1:10000 for WB
Mouse anti-FLAG M2	Sigma	RRID:AB_259529; F1804; 1:500 for IF
Rabbit anti-PHF3	Atlas Antibodies	RRID:AB_10600987; HPA025763; 1:500 for WB, 1:100 for IF
Rabbit anti-pS2 Pol II	Bethyl Laboratories	RRID:AB_519341; A300-654A; 1:1000 for WB
Rat anti-pS2 Pol II clone 3E10	Monocolonal antibody facility (Helmholtz Center, Munich)	80 μg/2x10^8 cells for ChIP
Rat anti-pS2 Pol II clone 3E10	Millipore	04-1571; 1:1000 for WB; 1:25 for IF
Mouse anti-pS5 Pol II clone 4H8	Cell Signaling	RRID:AB_2167468; 2629; 1:1000 for WB
Rat anti-pS5 Pol II clone 3E8	Monocolonal antibody facility (Helmholtz Center, Munich)	24 μg/1.2x10^8 cells for ChIP; 1:1000 for WB
Rat anti-pS5 Pol II clone 3E8	Millipore	04-1572; 1:1000 for WB; 1:25 for IF
Rat anti-pS7 Pol II clone 4E12	Millipore	RRID:AB_10618152; 04-1570; 1:1000 for WB; 10μg/10^8 cells for ChIP
Mouse anti-Pol II clone F-12	Santa Cruz	RRID:AB_630203; sc-55492; 15 μg/3x10^7 cells for ChIP; 5 μg/10^7 cells for IP; 1:1000 for WB, 1:200 for IF
Mouse anti-Pol II 8WG16	Abcam	ab817; 1:1000 for WB
Rabbit anti-TFIIF (RAP74)	Abcam	RRID:AB_2114552; ab28179; 1:1000 for WB
Rabbit anti-H3K27me3	Millipore	RRID:AB_310624; 07-449; 10 μg/3x10^7 cells for ChIP, 1:500 for IF
Mouse anti-TCEA1	Santa Cruz	sc-393439; 1:1000 for WB
Mouse anti-TCEA1	Abcam	ab185947; 10μg for 3x10^7 cells for ChIP
Mouse anti-α-tubulin clone B512	Sigma	RRID:AB_477582; T6074; 1:5000 for WB
Mouse anti-GFP clone 2B6	Egon Ogris	1:1000 for WB
Rabbit anti-GFP	Abcam	RRID:AB_303395; ab290; 1:1000 for WB; 6 μL/10^8 cells for ChIP, 1:1000 for IF
Rabbit anti-SPT6	Novus Biologicals	RRID:AB_2196402; NB100-2582; 1:1000 for WB
Mouse anti-DSIF	Becton Dickinson	RRID:AB_398420; 611107; 1:1000 for WB
Rabbit anti-PAF1	Abcam	RRID:AB_2159769; ab20662; 1:1000 for WB
Rabbit anti-SPT16	Santa Cruz	RRID:AB_661341; sc-28734; 1:1000 for WB

Mouse anti-SSRP1	Abcam	RRID:AB_449020; ab26212; 1:1000 for WB
Rabbit anti-β-Tubulin III TuJ1	Sigma	RRID:AB_262133; T2200; 1:500 for IF
Mouse anti-GFAP	Sigma	RRID:AB_477010; G3893; 1:500 for IF
Goat anti-rabbit-IgG-HRP	Jackson ImmunoResearch	111-035-008; 1:10000 for WB
Goat anti-rat-IgG-HRP	Jackson ImmunoResearch	112-035-008; 1:10000 for WB
Goat anti-mouse-IgG-HRP	Jackson ImmunoResearch	115-035-008; 1:10000 for WB
Goat anti-mouse Alexa Fluor 488	Invitrogen	A11001; 1:500 for IF
Goat anti-rabbit Alexa Fluor 488	Invitrogen	A11008; 1:500 for IF
Goat anti-mouse Alexa Fluor 568	Invitrogen	A11004; 1:500 for IF
Goat anti-rabbit Alexa Fluor 568	Invitrogen	A11011; 1:500 for IF
Goat anti-rat Alexa Fluor 594	Invitrogen	A11007; 1:500 for IF

Supplementary Table 4. Oligonucleotides

	Sequence
PHF3 KO gRNA	5’-TGATACTAGTACTTTTGGAC-3’
Phf3 KO gRNA	5’-ATTCGGGTTCCTCGGCCGTC-3’
PHF3-EGFP tagging gRNA1	5’-CAGTGTGGTCCCTATCTTTG-3’
PHF3-EGFP tagging gRNA2	5’-TAAAATTTGCAGGCTGCTTC-3’
PHF3 ΔSPOC gRNA1	5’-TGGCTCGATTGAACTTCATC-3’
PHF3 ΔSPOC gRNA2	5’-GGTCCATCAAAAGGCACAAG-3’
Repair template for ΔSPOC	5’-TTAGGCTTTTTAATGTCATTTTCTAGTCCAGAGATGCCTGGAACTGTTGAAGTTGAGTCTACCTTTCTGGCTCGAGTGCCTTTTGATGGACCTGGTAGGTATACGTTTTAATAATAGGATAAGAATGAAATAACATGGGAGGTGGGACCA-3’
3’ RNA adaptor	5'- 5Phospho rNrNrNrNrNrNrNrNrGrArUrCrGrUrCrGrGrArCrUrGrUrArGrArArCrUrCrUrGrArArC-/inverted dT/ -3'
5’ RNA adaptor	5'- rCrCrUrUrGrGrCrArCrCrCrGrArGrArArUrUrCrCrArNrNrNrN -3'
INA TSS FW	5’-AAGTTTGCCAACCTGAACGA-3’
INA TSS RV	5’-CTCTCCAAGGACTCGTTGGC-3’
INA body FW	5’-CAGAGCTGCCAGCTGAATTG-3’
INA body RV	5’-AGACCATGTCTGGATGACGC-3’
INA 3’UTR FW	5’-GTCCACACCCAATTCCACTC-3’
INA 3’UTR RV	5’-TACCCACTGAGCAGAGCGCG-3’
GPR50 TSS FW	5’-TCTGCGCGATGGTTATCACC-3’
GPR50 TSS RV	5’-CAGAATTCCGGAGCTTCTTG-3’
GPR50 body FW	5’-TCAGCCCTACAAGACCGGAG-3’
GPR50 body RV	5’-GTTACATGCCGCGTCCACGT-3’
GPR50 3’UTR FW	5’-CTACATCCACCTTTCAGGCG-3’
GPR50 3’UTR RV	5’-CTGAGGACTGAAGGTCGACC-3’
CTRL FW	5’-AGCTGTACTGTGGCACAGAA-3’
CTRL RV	5’-AGGTGATGGATATGTTCGCT-3’
GAPDH FW	5’-CGGGAAGCTTGTCATCAA-3’
GAPDH RV	5’-CGCCAGTGGACTCCACGA-3’
INA FW	5’-CACAGTGCCGAGGTAGCTGG-3’
INA RV	5’-GTGGCGTGCCATCTCACTCT-3’

GPR50 FW	5'-CAAGAAGCTCCGGAATTCTG-3'
GPR50 RV	5'-TAACTGGCTCAGATCCCAGC-3'
mGapdh FW	5'-CAATGTGTCCGTCGTGGATC-3'
mGapdh RV	5'- AAGTCGCAGGAGACAACCTG-3'
mPhf3FW	5'- GCAGAGAGTCTAGCAGATGC-3'
mPhf3 RV	5'- CAGTTCCAGGCATCTCTGGC-3'
mOct4 FW	5'- GTGGACCTCAGGTTGGACTGG-3'
mOct4 RV	5'- CTTCTGCAGGGCTTTCATGTC-3'
mNanog FW	5'- AGCAGATGCAAGAACTCTCCTC-3'
mNanog RV	5'- AATGGATGCTGGGATACTCCAC-3'
mSox2 FW	5'- ACAGCATGATGCAGGAGCAG-3'
mSox2 RV	5'- GTCTGCGAGCTGGTCATGGA-3'
mSox21 FW	5'-CAGGAGAACCCTAAGATGCA-3'
mSox21 RV	5'-GATGCTCCTTCATGTGCATG-3'
mPou3f2 - Brn2- Oct7 FW	5'- TCAGACGACCTGGAGCAGTT-3'
mPou3f2 - Brn2- Oct7 RV	5'- ACCTGCAGATGGTGGTCTGC-3'
mAscl1 FW	5'- CAACTGGTTCTGAGGACCTG-3'
mAscl1 RV	5'- CCCTGTAGGTTGGCTGTCTG-3'
mNes FW	5'- AGAGAGGCGCTGGAACAGAG-3'
mNes RV	5'- CCCACCTTCCAGGATCTGAG-3'
mPou3f1 - Oct6 FW	5'- CTCACCTTTTCTCCGGGC-3'
mPou3f1 - Oct6 RV	5'- ATACACAGATGCGGCTCT-3'
mFgf5 FW	5'- CTGTGTCTCAGGGGATTGTAGG-3'
mFgf5 RV	5'- GATCGCGGACGCATAGGTATTA-3'
mOtx2 FW	5'- CTCCTGGAGGAGAGAGCAGTC-3'
mOtx2 RV	5'- CTTGGTGGGTAGATTTGGAGTGA-3'
mPax6 FW	5'- CACCATGGCAAACAACCTGC-3'
mPax6 RV	5'- TGTTGAAGTGGTCCCCGAGG-3'
SLAM-seq INA FW	5'- CACGACGCTCTTCCGATCTNNNNNNTCTGTCCAGCAGTCACTTCG-3'
SLAM-seq Oligo-dT	5'-GTTCAGACGTGTGCTCTTCCGATCT-(T)n-V-3'
Illumina FW	5'- AATGATACGGCGACCACCGAGATCTACACTCTTTCCCTACACGACGCTC TTCCGATCT-3'
Illumina RV	5'- CAAGCAGAAGACGGCATACGAGATNNNNNNGTGACTGGAGTTCAGACG TGTGCTCTTCCGATCT-3'
EC-phf3-nontemplate	5'- GGCAGTACTAGTATTCTAGTATTGACAGTACTTGAGCTTGATC-3'
EC-phf3-template	5'- GATCAAGCTCAAGTACTGTAGCCTGGTCTATACTAGTACTGCC-3'
RNA50	5'- rUrArArCrGrArGrArUrCrArUrArArCrArUrUrUrGrArArCrArArGrArArUrArUrA rUrArUrArCrArUrArArArGrArCrCrArGrGrC-3'
Arrest-nontemplate	5'- GGTTGTTGAAGTTATGGAGTAGGGTAATATATCAACGGTGGTATATCCA GTGATTTTTTTCTCCATTTTAGCTTCCT-3
Arrest-template	5'- AGGAAGCTAAAATGGAGAAAAAAATCACTGGATATACCACCGTTGATA TATTACCCTACTCCATAACTTCAACAACC-3'
5'-FAM-Arrest-RNA	5'- /56-FAM/rUrArUrArCrArUrArArUrGrGrArGrUrArGrGrGrU-3'

SUPPLEMENTARY FIGURES

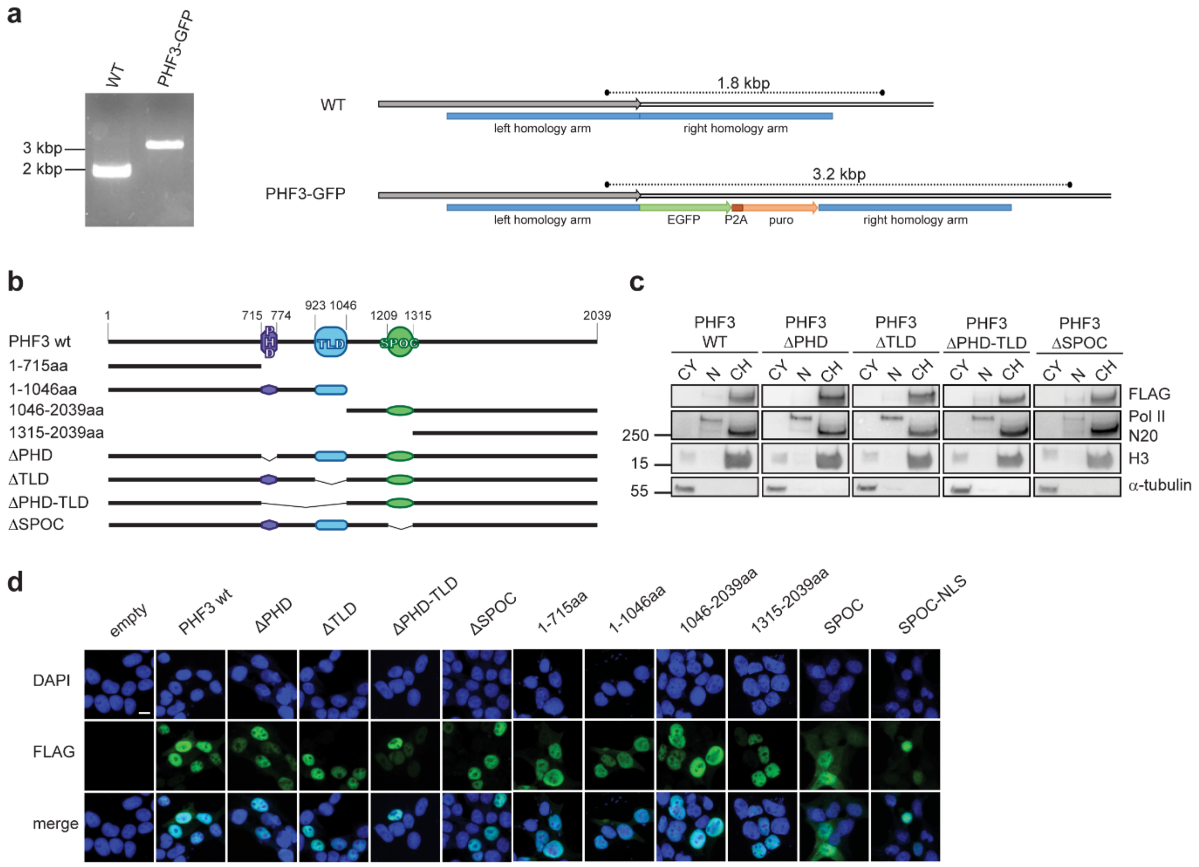

Supplementary Fig. 1. Generation of the PHF3-GFP cell line and localization of PHF3 truncation mutants. a, CRISPR/Cas9 strategy and PCR validation of endogenous tagging of PHF3 with GFP at the C-terminus. The experiment was performed once. **b,** A scheme of PHF3 truncation constructs used in (C-D). **c,** Chromatin localization of FLAG-PHF3 truncation mutants revealed by biochemical fractionation and Western blotting. Alpha-tubulin, Pol II and H3 were used as markers of cytoplasmic (CY), nucleoplasmic (N) and chromatin (CH) fractions, respectively. The experiment was performed once. **d,** Nuclear localization of FLAG-PHF3 truncation mutants revealed by immunofluorescence staining with anti-FLAG and nuclei counterstaining with DAPI. Scale bar=10 µm. The experiment was performed once.

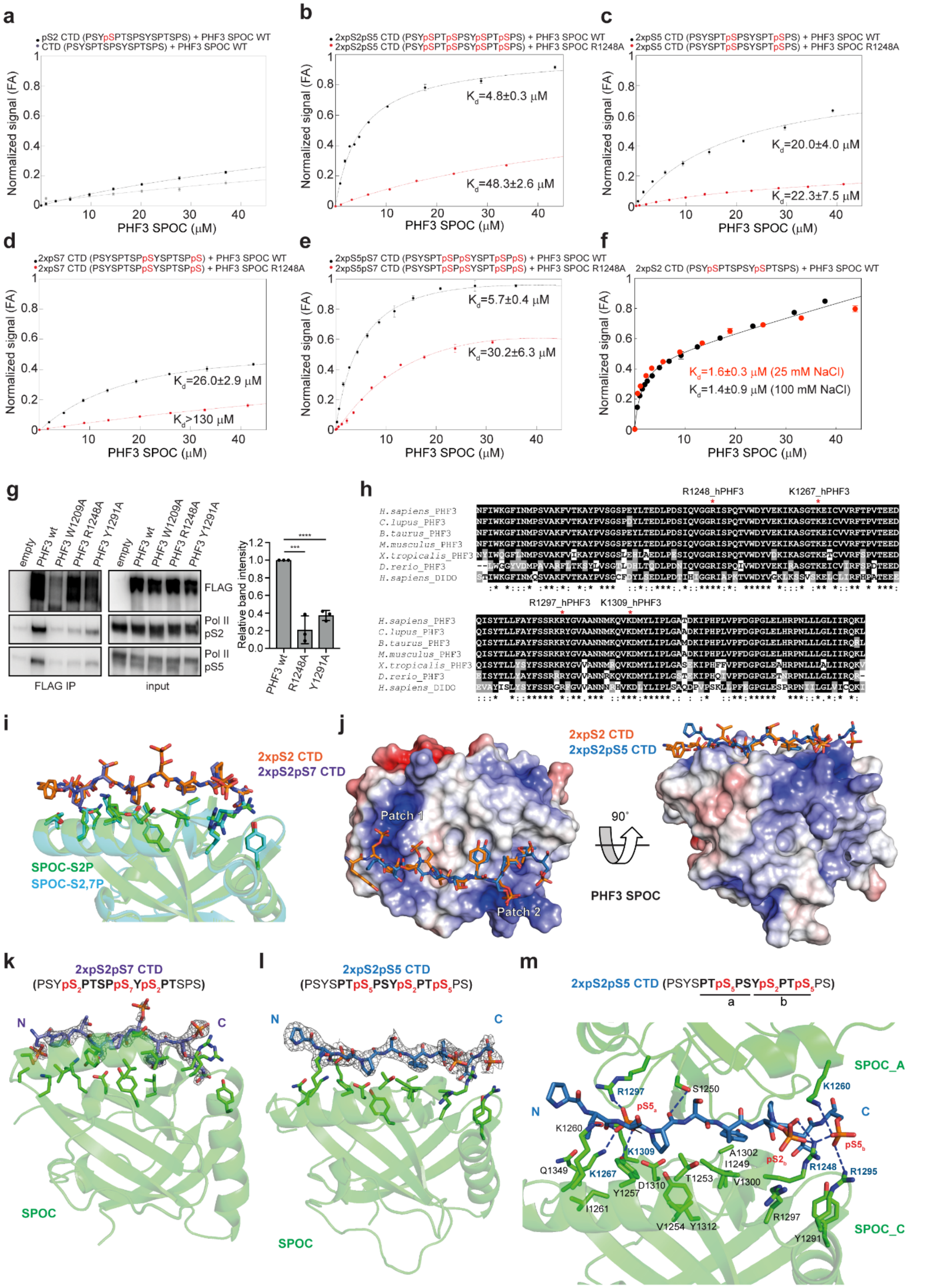

Supplementary Fig. 2. Analysis of interaction between PHF3 SPOC and CTD peptides. a-e, Fluorescence anisotropy (FA) measurement of the binding of **a,** pS2 on one CTD repeat and non-phosphorylated CTD, **b,** 2xpS2pS5, **c,** 2xpS5, **d,** 2xpS7 and **e,** 2xpS5pS7 CTD peptides to PHF3 SPOC WT (black) or R1248A mutant (red). All experiments were performed in a buffer with 25 mM NaCl. Normalized fluorescence anisotropy is plotted as a function of protein concentration (n=3). The data were normalized for visualization purposes and the experimental isotherms were fitted to one site saturation with non-specific binding model. **f,** Fluorescence anisotropy measurement of the binding of 2xpS2 to PHF3 SPOC WT at 25 mM NaCl (red) or 100 mM NaCl

(black). The salt concentration does not have an effect on the binding affinity. **g,** Mutation of conserved residues in PHF3 SPOC shows loss of interaction with Pol II. W1209 is important for structural integrity; R1248 and Y1291 were shown to mediate SHARP SPOC binding to SMART and NCoR peptides[1]. The bar graph shows quantification of Pol II in the eluates, normalized to the immunoprecipitated PHF3 bait. Three biologically independent experiments were performed. Data are presented as mean values +/- standard deviation. One-tailed, two-sample equal variance t-test was used to determine p-values. P-values are indicated in Supplementary Data 7. **h,** Multiple sequence alignment of PHF3 SPOC from *Homo sapiens*, *Canis lupus*, *Bos taurus*, *Mus musculus*, *Xenopus tropicalis* and *Danio rerio*, and DIDO SPOC from *Homo sapiens*. Four basic residues shown to bind 2xpS2 CTD in PHF3 SPOC are indicated with a red asterisk. **i,** Overlay of SPOC co-structures with 2xpS2 and 2xpS2pS7 CTD peptides shows a similar extended conformation. **j,** Overlay of PHF3 SPOC structures with 2xpS2 and 2xpS2pS5 shows a difference in conformation of the N-terminal CTD residues. The color coded electrostatic surface potential of SPOC was drawn using the Adaptive Poisson-Boltzmann Solver package within PyMol. The electrostatic potential ranges from −5 (red) to +5 (blue) kT/e. **k,** $2\ F_o - F_c$ electron density map of 2xpS2pS7 peptide contoured at the 1.5σ level. **l,** $2\ F_o - F_c$ electron density map of 2xpS2pS5 peptide contoured at the 1.5σ level. **m,** Hydrogen bonding interactions between 2xpS2pS5 and two PHF3 SPOC molecules. SPOC residue labels from two positively charged patches are colored blue.

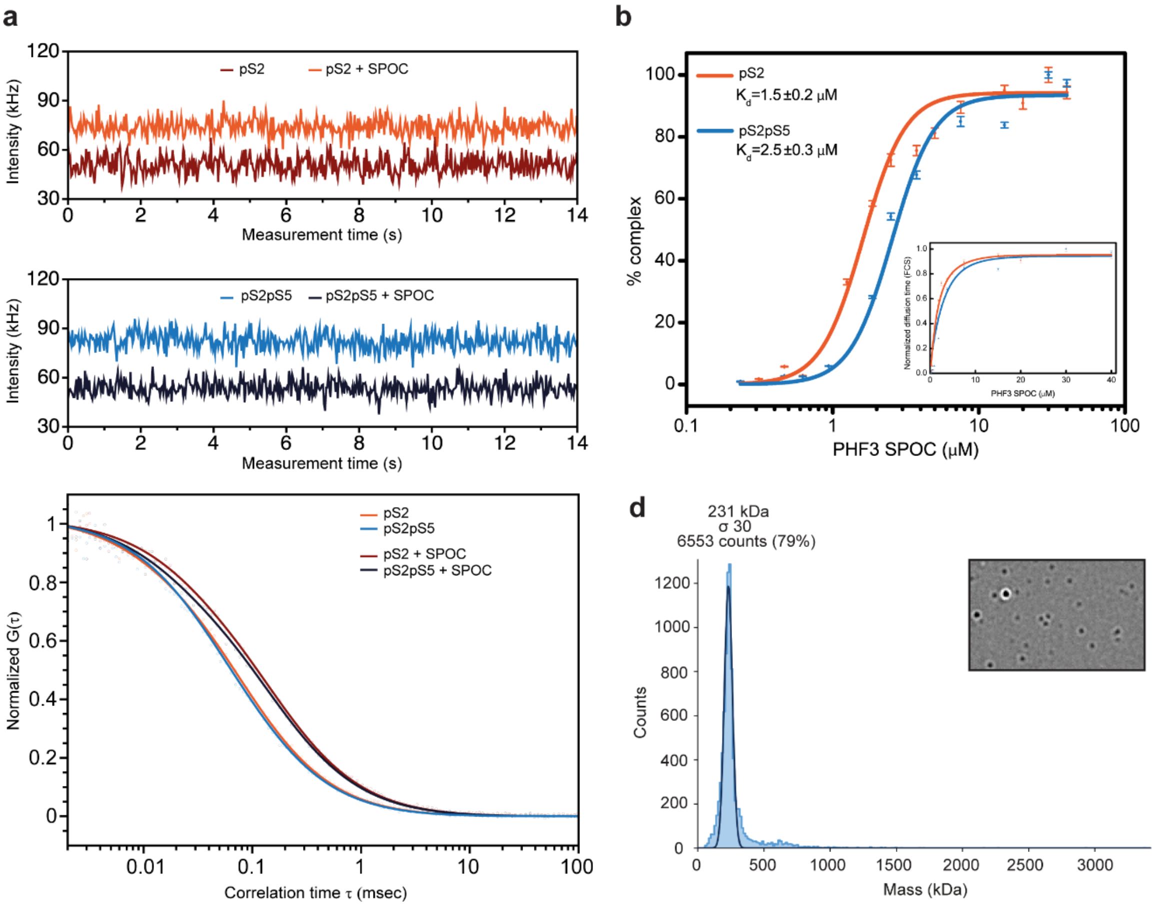

c

	diffusion time* τ_D [μsec]	diffusion coefficient D_{trans} [μm²/sec]	hydrodynamic radius [Å]	molecular weight FCS [kDa]	molecular weight predicted [kDa]
PHF3 SPOC	127.6 ± 8.0	111.6 ± 6.6	19.2 ± 1.2	17.9 ± 3.6	17.9
Peptide pS2 CTD	74.4 ± 2.7	191.3 ± 6.7	9.2 ± 0.3§	1.9 ± 0.2§	1.8
Peptide + 40 μM SPOC	133.0 ± 7.8	107.1 ± 6.0	20.0 ± 1.2	20.3 ± 3.8	19.7

* 35 μm pinhole size   § assuming ellipsoidal shape

	diffusion time* τ_D [μsec]	diffusion coefficient D_{trans} [μm²/sec]	hydrodynamic radius [Å]	molecular weight FCS [kDa]	molecular weight predicted [kDa]
PHF3 SPOC	127.6 ± 8.0	111.6 ± 6.6	19.2 ± 1.2	17.9 ± 3.6	17.9
Peptide pS2pS5 CTD	75.0 ± 0.9	189.8 ± 2.2	9.2 ± 0.1§	2.0 ± 0.1§	2.0
Peptide + 40 μM SPOC	127.0 ± 5.0	112.1 ± 4.2	19.1 ± 0.8	17.7 ± 2.2	19.8

* 35 μm pinhole size   § assuming ellipsoidal shape

Supplementary Fig. 3. Analysis of binding stoichiometry between PHF3 SPOC and CTD peptides by fluorescence correlation spectroscopy (FCS) and analysis of PHF3 oligomeric state by mass photometry. a, Fluorescence intensity fluctuations of all the molecules through a Gaussian confocal volume of 0.65 femtolitres are recorded over 15 seconds. Normalized autocorrelation function G(τ) describes the observed fluctuations of the fluorescence intensity from the mean intensity at any time compared to fluctuations at delayed time. Diffusion parameters (τ_D and D_{trans}) are obtained from the normalized autocorrelated dataset using a Marquardt nonlinear least-square fitting routine. **b,** Percentage of SPOC-CTD peptide complex formation calculated from the change in translational diffusion behavior. K_d values of 2xpS2 and 2xpS2pS5 CTD peptides binding to SPOC are obtained from normalized translational diffusion times (inset). **c,** Biophysical parameters of SPOC, 2xpS2 and 2xpS2pS5 peptides and their complexes obtained by FCS (T=20°C). **d,** Mass distribution and representative ratiometric image of 20nM PHF3.

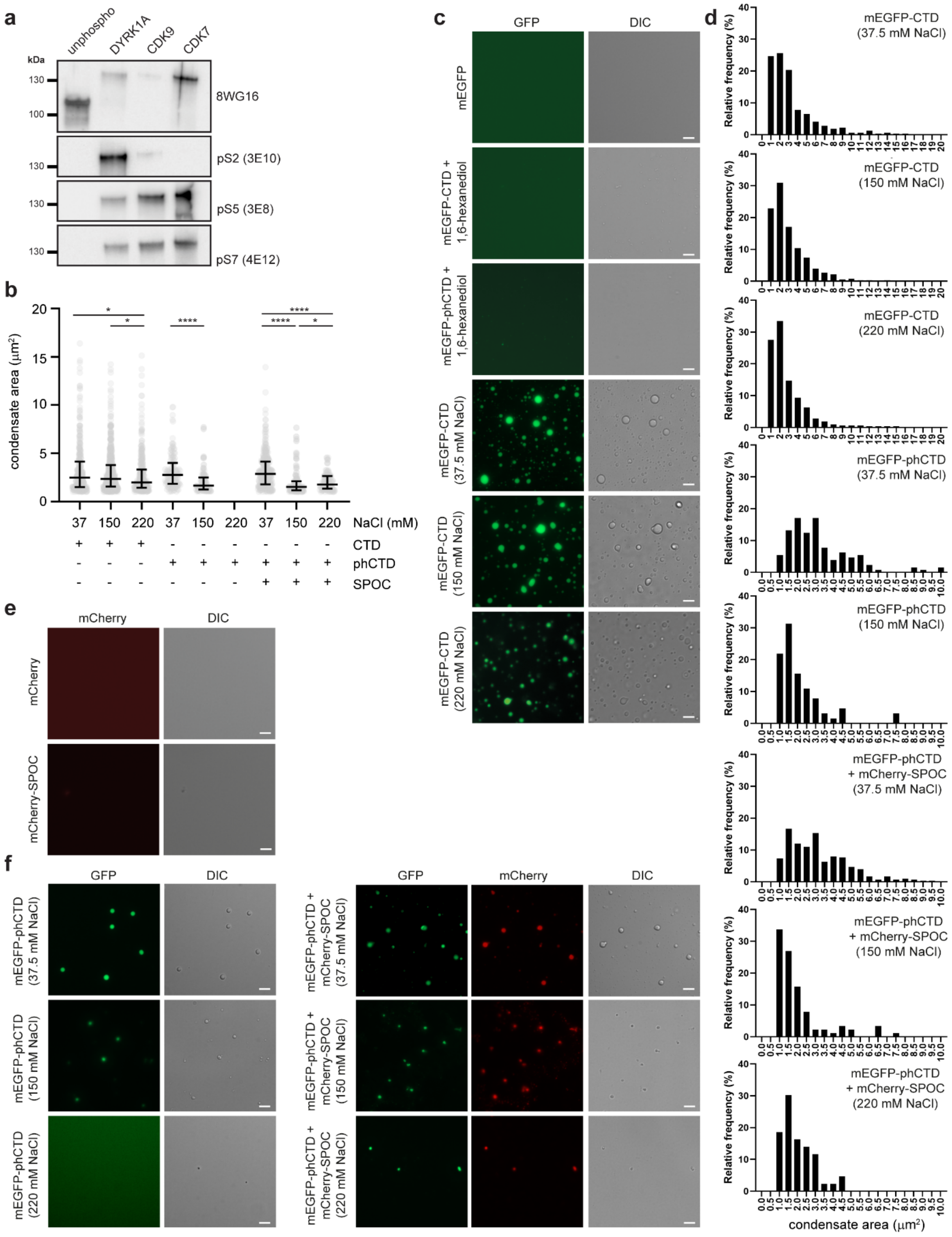

Supplementary Fig. 4. ***In vitro*** **Pol II CTD LLPS assays. a,** Western blot analysis of phosphorylation of purified mEGFP-CTD with DYRK1A, CDK9 and CDK7. 2.2 pmol EGFP-CTD were loaded per lane, membranes were probed with antibodies against unphosphorylated CTD (8WG16), pS2, pS5 and pS7. The experiment was performed twice. **b,** Quantification of condensate area (μm^2) for condensates from panels **c** and **f**. N=320 (CTD 37.5); 556 (CTD 150); N=321 (CTD 220); 129 (phCTD 37.5); 64 (phCTD 150); 302 (phCTD+SPOC 37.5); 89 (phCTD+SPOC 150); 129 (phCTD+SPOC 220). Data are presented as median with interquartile range. Mann-Whitney test was used to determine p-values (Supplementary Data 7). **c,** Representative images of *in vitro* LLPS assays with 5μM unphosphorylated mEGFP-CTD at different salt concentrations. 5μM mEGFP does not form condensates. Addition of 10% 1,6-hexanediol abrogates CTD condensation. Scale bar = 5μm. **d,** Frequency

distribution analysis of condensates shown in panels **c** and **f**. 200-300 condensates were analysed. **e,** 5µM mCherry and 5µM mCherry-SPOC do not form condensates. **f,** Representative images of *in vitro* LLPS assays with 5µM DYRK1A-phosphorylated mEGFP-CTD without or with 5µM mCherry-SPOC at different salt concentrations. Scale bar = 5µm. The experiments were repeated three times and representative images are shown in **c, e, f**.

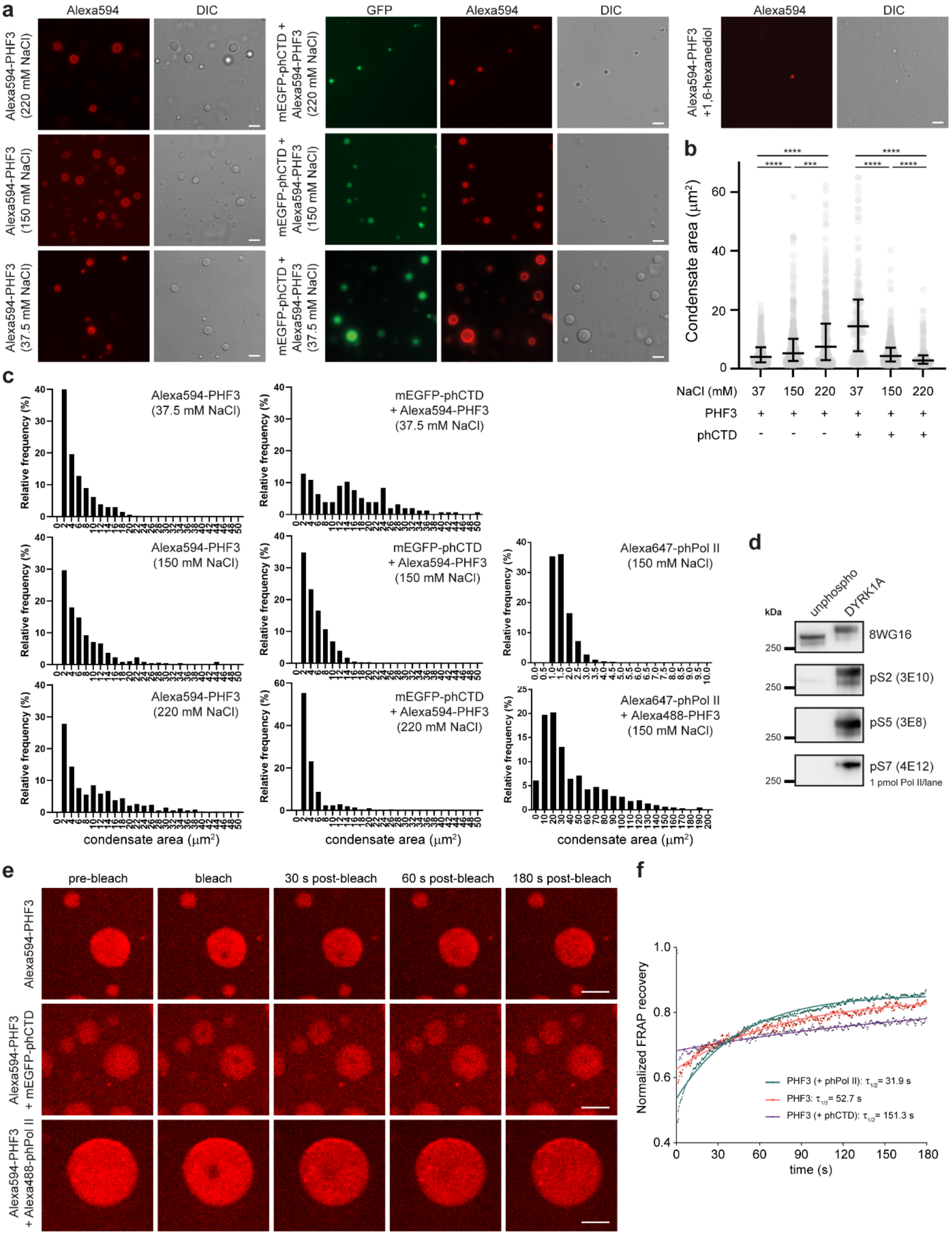

Supplementary Fig. 5. ***In vitro*** **PHF3 LLPS assays. a,** Representative images of *in vitro* LLPS assays with 3μM Alexa594-PHF3 and 5μM DYRK1A-phosphorylated mEGFP-CTD. Scale bar=5μm. The experiments were repeated three times and the representative images are shown. **b,** Quantification of condensate area (μm^2) for >150 condensates per condition from panel **a**. **c,** Frequency distribution analysis of condensates shown in panel **a** and Fig. 3a. N=337 (PHF3 37.5); 480 (PHF3 150); N=350 (PHF3 220); 167 (PHF3 + phPol II 37.5); 580 (PHF3 + phPol II 150); 208 (PHF3 + phPol II 220). Data are presented as median with interquartile range. Mann-Whitney test was used to determine p-values (Supplementary Data 7). **d,** Western Blot analysis of phosphorylation of purified Pol II with DYRK1A. 1 pmol Pol II was loaded per lane. The experiment was performed twice. **e-f,** FRAP

analysis of PHF3 condensates without or with phCTD and phPol II. 3µM Alexa594-PHF3, 3µM Alexa594-PHF3 + 5µM DYRK1A-phosphorylated mEGFP-CTD, 1.5µM Alexa594-PHF3 + 1.5µM DYRK1A-phosphorylated Alexa488-Pol II were used. **e,** Representative images of Alexa594-PHF3 before bleaching, upon bleaching and at different time points after bleaching. Scale bar = 5µm. **f,** Relative quantification of fluorescence recovery kinetics of PHF3 condensates following partial bleaching (N=19-21 condensates per condition). The FRAP curves were fitted with an exponential one phase association model [Y=$Y0$ + (Plateau-$Y0$)*(1-exp(-K*x))]. $\tau_{1/2}$: the time when half of the fluorescent signal was recovered after FRAP. Fitting parameters for PHF3: $Y0$= 0.6264, Plateau = 0.8461, K = 0,01316, τ = 75.97, $\tau_{1/2}$ = 52.7, R^2 = 0.9497; PHF3 + phCTD: $Y0$= 0.6824, Plateau = 0.8577, K = 0.004582, τ = 218.2, $\tau_{1/2}$ = 151.3, R^2 = 0.92; PHF3 + phPolII: $Y0$= 0.5351, Plateau = 0.8548, K = 0.02176, τ = 45.96, $\tau_{1/2}$ = 31.9, R^2 = 0.9867.

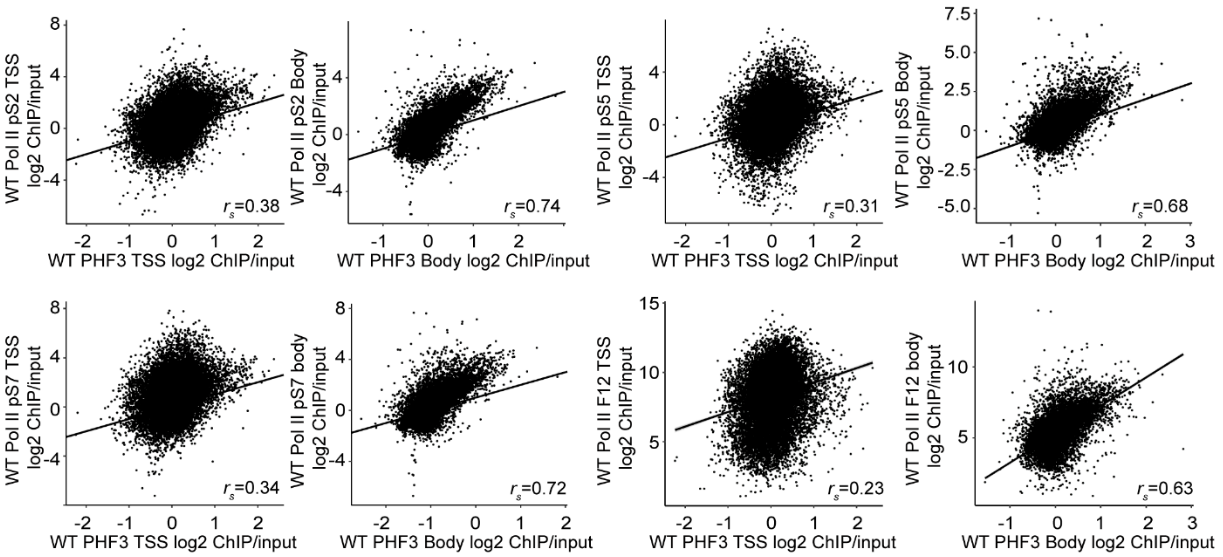

Supplementary Fig. 6. PHF3 travels with Pol II. Scatter plots showing Pol II pS2, pS5, pS7 and F-12 occupancy relative to PHF3 occupancy on transcription start sites (TSS) and gene body. Spearman's correlation coefficients are indicated.

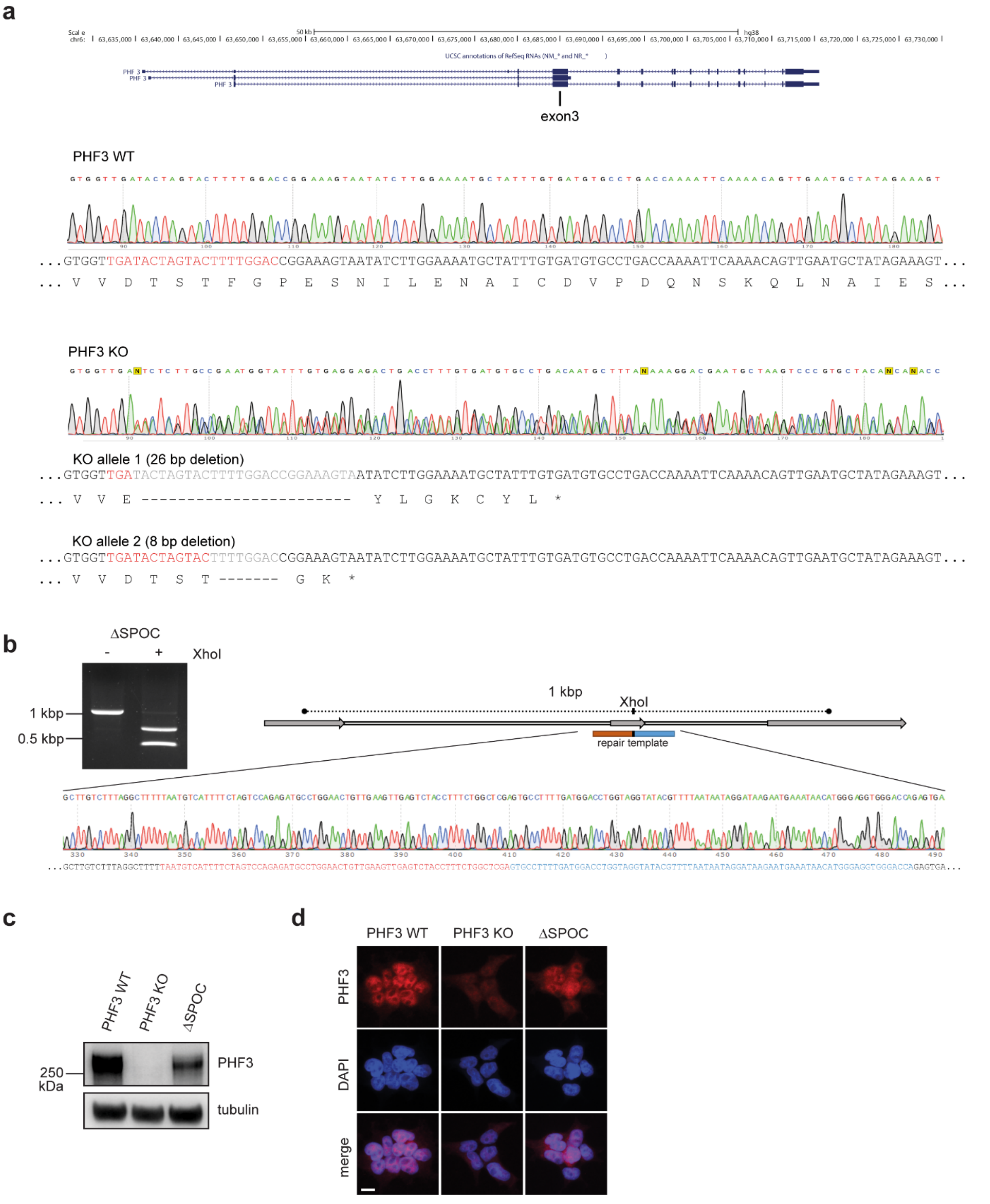

Supplementary Fig. 7. Generation of HEK293T PHF3 knock-out (KO) and ΔSPOC cells by CRISPR/Cas9. a, Sequencing snapshots and sequence reconstruction of human PHF3 KO in HEK293T cells generated by a frameshift-induced stop codon in exon 3. gRNA target region is shown in red, deleted regions are in grey. **b,** Strategy and validation of endogenous deletion of the SPOC domain from PHF3 (ΔSPOC) by PCR and sequencing. **c,** Western blot and **d,** immunofluorescence analysis of PHF3 KO and ΔSPOC in HEK293T cells. Scale bar=10 µm. The experiments in **b,c,d** were performed once.

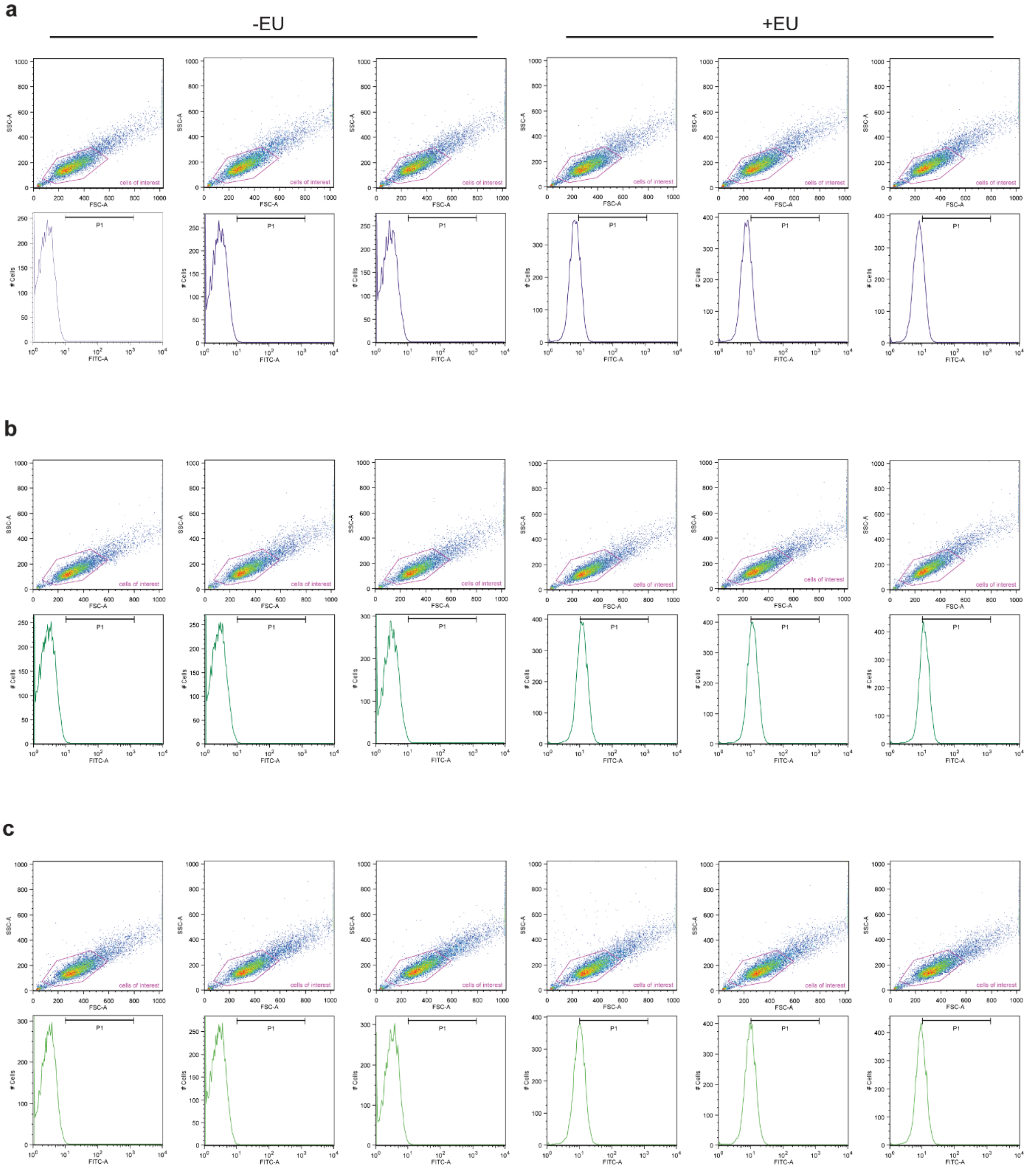

Supplementary Fig. 8. Gating strategy for the graphs in Fig. 4d,e. a, -EU triplicates (left) and +EU triplicates (right) graphs for the HEK293T WT cells are shown. The whole cell population is shown in the top row, with the gated population of interest. The gated cells of interest were then analysed on the basis of their FITC signal to quantify EU incorporation (bottom row histogram). The P1 gating used for statistical analysis is also shown. **b,c,** As in a, for PHF3 KO and dSPOC cells, respectively.

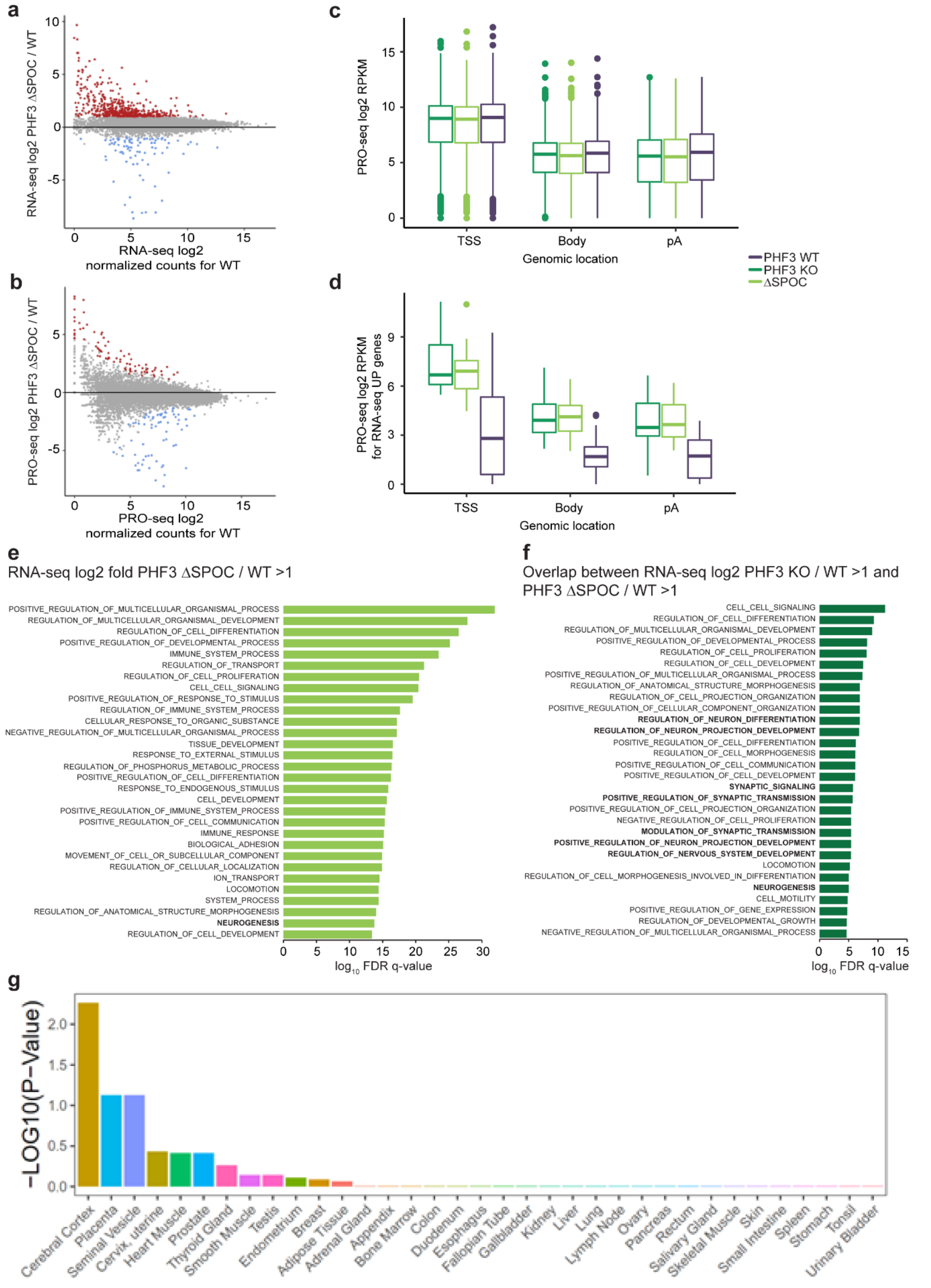

Supplementary Fig. 9. Derepression of neuronal genes in PHF3 KO and ΔSPOC cells. a, RNA-seq analysis shows upregulation of 638 genes (red dots, fold-change>2, p<0.05) and downregulation of 74 genes (blue dots, fold-change>2, p<0.05) in PHF3 ΔSPOC compared to WT. Drosophila S2 cells were used for spike-in normalization. **b,** PRO-seq analysis shows upregulation of 78 genes (red dots, fold-change>2, p<0.05) and downregulation of 70 genes (blue dots, fold-change>2, p<0.05) in PHF3 ΔSPOC compared to WT. Drosophila S2

nuclei were used for spike-in normalization. **c,d,** Box plot quantification of PRO-seq reads on transcription start sites (TSS), gene body and polyadenylation sites (pA) in PHF3 WT, KO and ΔSPOC cells for **c,** all genes or **d,** RNA-seq upregulated genes. KO and ΔSPOC show statistically significant difference compared to WT for all conditions based on the Wilcoxon signed-rank test (p-value$<10^{-5}$, N~30 000 genes). Statistics are indicated in detail in Supplementary Data 7. **e,f,** GO analysis of genes upregulated in PHF3 ΔSPOC and genes upregulated in both PHF3 KO and ΔSPOC shows enrichment of genes involved in neurogenesis. GSEA Biological processes tool was used. **g,** TissueEnrich analysis shows the enrichment of cerebral cortex among transcriptionally upregulated genes in PHF3 KO. TissueEnrich analyses the enrichment of a particular gene set in the tissue specific expression profiles provided by the GTEx transcriptional compendium (https://www.gtexportal.org/home/faq#citePortal). The y-axis shows the -log10 of Benjamini-Hochberg corrected p-value.

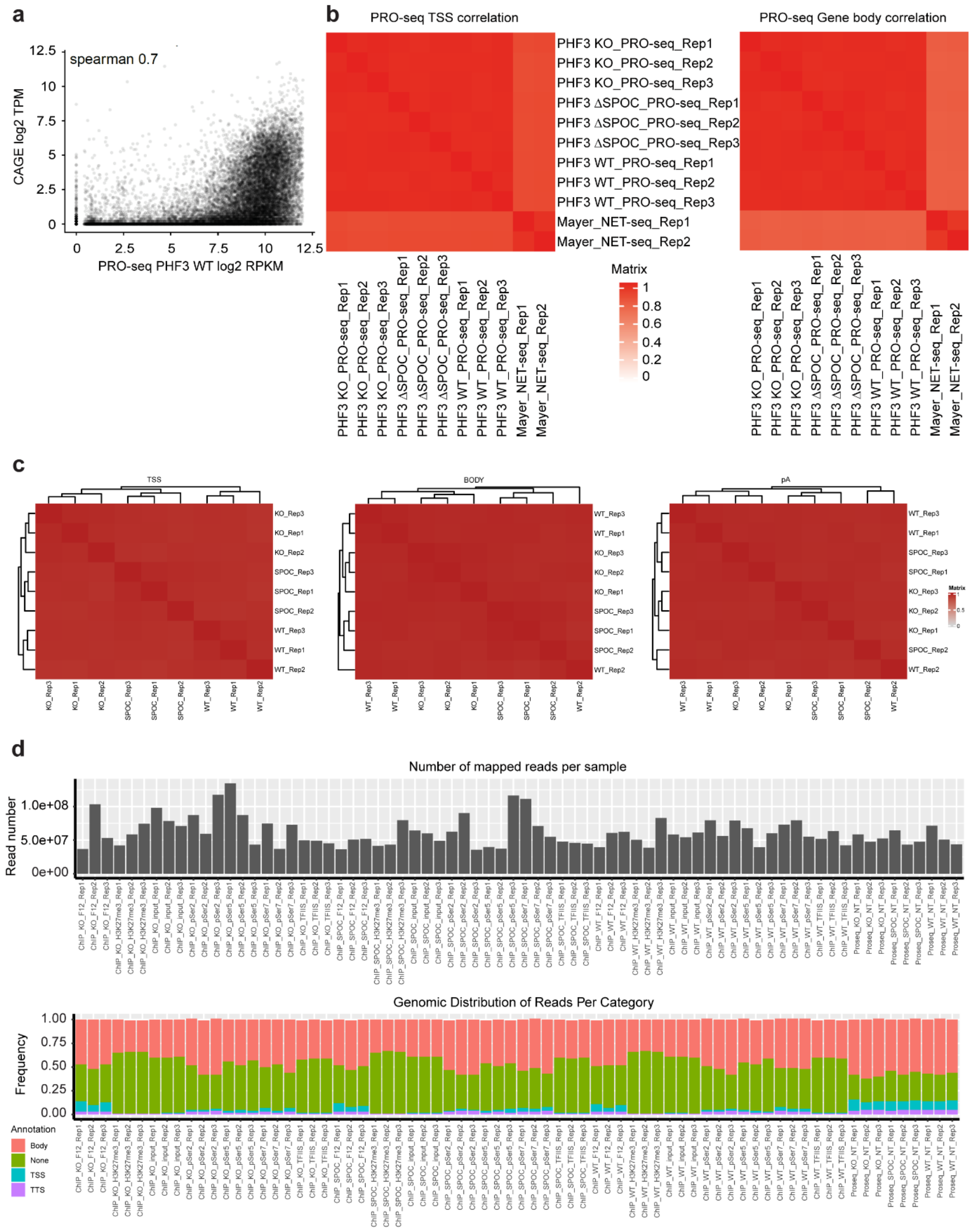

Supplementary Fig. 10. Validation of PRO-seq data analysis. a, Comparison between our PRO-seq PHF3 WT and CAGE data (TPM stands for transcripts per million, RPKM stands for reads per kilobase million). **b,** Comparison between our PRO-seq data for PHF3 WT and KO, and published NET-seq data from HEK293 cells. **c,** Correlation matrix for PRO-seq replicates. The mean Pearson correlation coefficient between the samples was 0.96, ranging from 0.95-0.97 for different gene parts (transcription start sites, gene body and polyadenylation sites). **d,** Distribution of ChIP-seq and PRO-seq data along the functional genomic features showing the absolute number of mapped reads (above) and the frequency of reads in different categories.

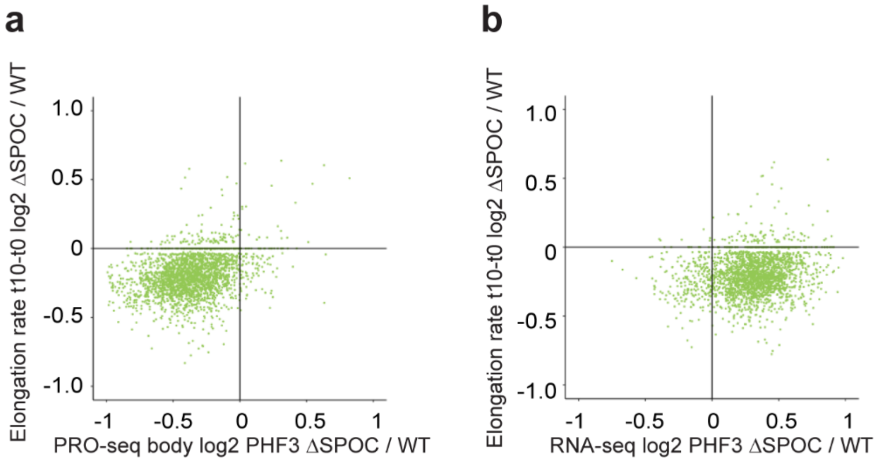

Supplementary Fig. 11. a, Relationship between PRO-seq body fold change and t10 elongation rate (10 min after DRB washout) fold change for PHF3 ΔSPOC vs WT. **b,** Relationship between RNA-seq fold change and t10 elongation rate (10 min after DRB washout) fold change for PHF3 ΔSPOC vs WT.

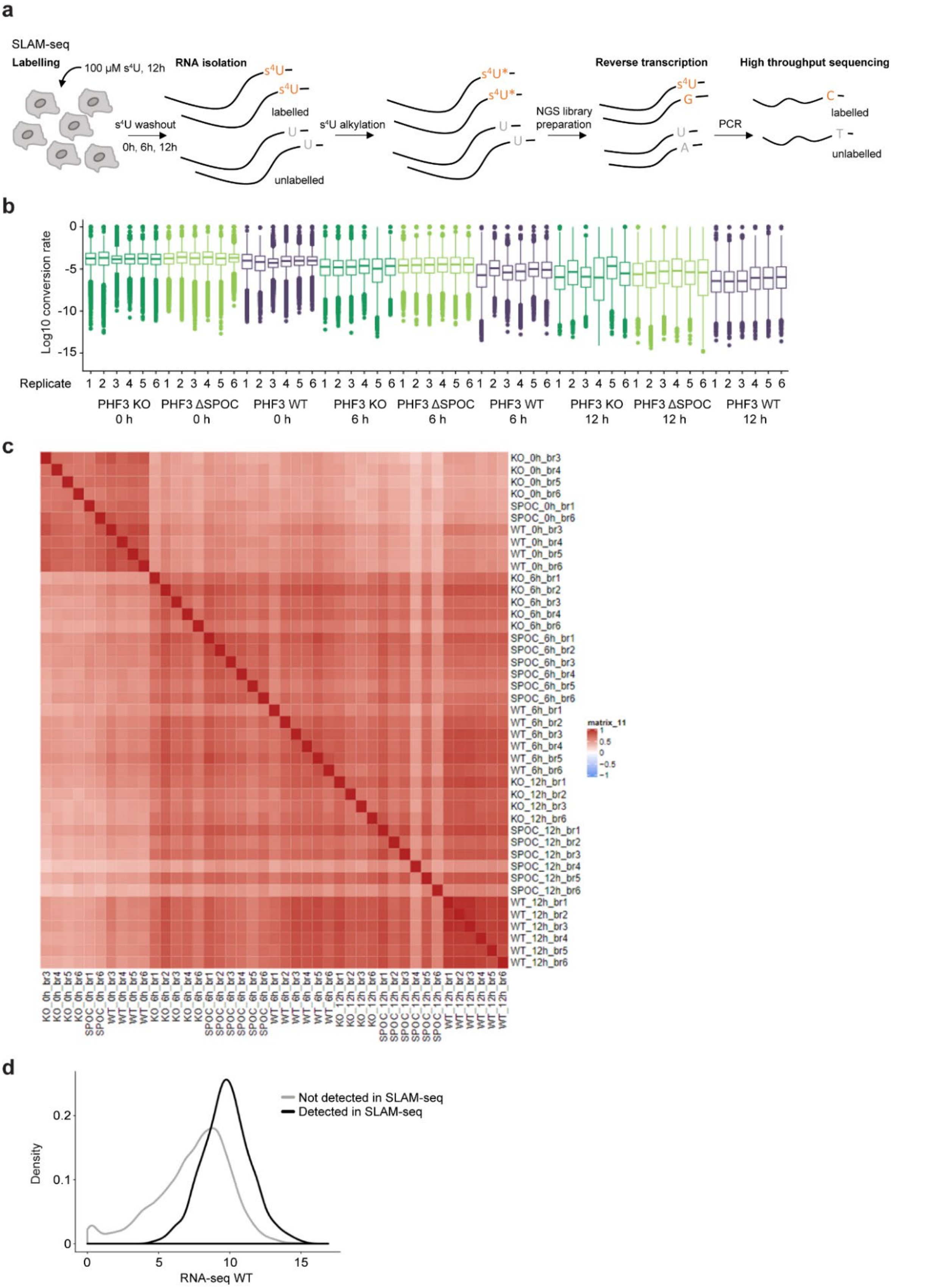

Supplementary Fig. 12. SLAM-seq analysis of mRNA stability in PHF3 WT, KO and ΔSPOC cells. a, Schematics for SLAM-seq. **b,** Conversion rate distributions per replicate for each genotype and condition. '0 h' corresponds to 12 hours of s^4U labelling, '6 h' and '12 h' correspond to chase with uridine (N=757 genes). **c,** Conversion rate correlation matrix. Median Spearman correlation coefficient of conversion rates for replicate samples belonging to the same group (same genotype and timepoint) was 0.75. **d,** SLAM-seq captures mid/high expressed genes but not low-expressed genes.

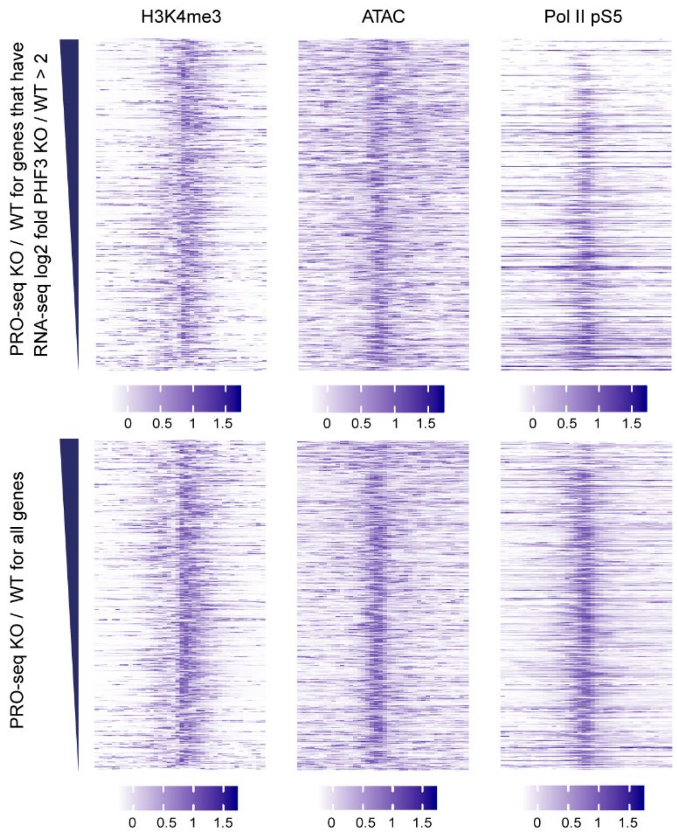

Supplementary Fig. 13. Promoter properties of genes that were upregulated in PHF3 KO and ΔSPOC. Genes upregulated in PHF3 KO are enriched for H3K27me3 repressive mark but also exhibit features of open promoters such as H3K4me3, ATAC signal and Pol II pS5. The heatmaps show ChIP signal in the region of +/- 2kb around TSS. ATAC-seq data was from ArrayExpress ID: E-MTAB-6195; H3K4me3 data was from ENCODE ID: ENCSR000DTU. Prior to visualization, each signal in each TSS was scaled by dividing the corresponding values with the range (max value - min value). This was done for each experiment separately, in order to enable visualization of ChIP data on completely different scales.

a

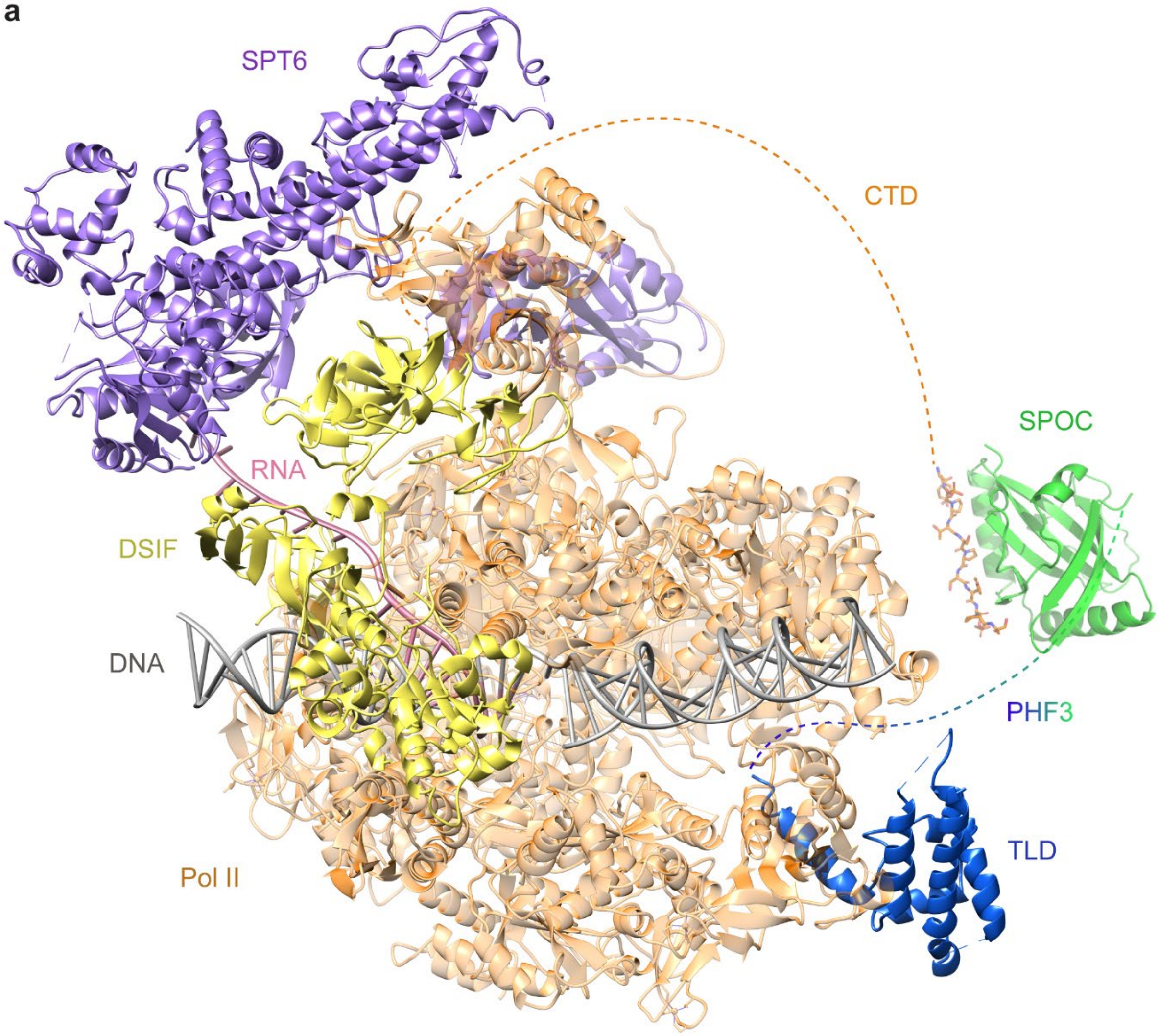

b

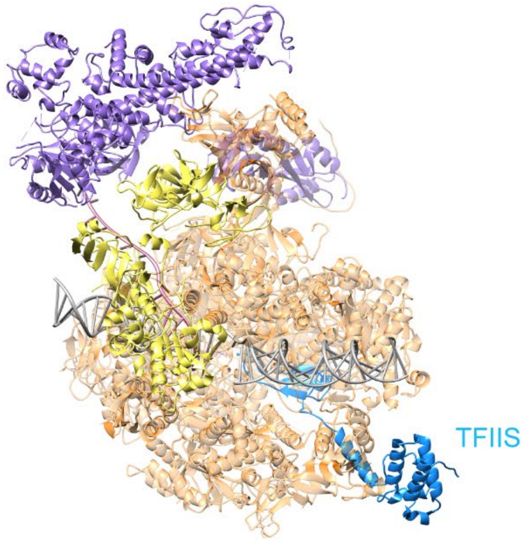

Supplementary Fig. 14. A structural model of PHF3 TLD-SPOC binding to Pol II and regulating backtracking. a, Yeast Bye1 TLD (4BY7) shown in blue was overlayed onto transcribing bovine Pol II (6GMH) shown in orange with elongation factors SPT6 (purple) and DSIF (yellow). PHF3 SPOC (green) in complex with 2xS2P CTD (orange) (6IC8) is connected with TLD and Pol II with dashed lines due to the absence of structural data for the disordered CTD and the linker between TLD and SPOC. **b,** TFIIS occupies a similar location on Pol II (5IYB) as Bye1/PHF3 TLD. TFIIS also has a domain III, which inserts itself into the Pol II active center to stimulate RNA cleavage.

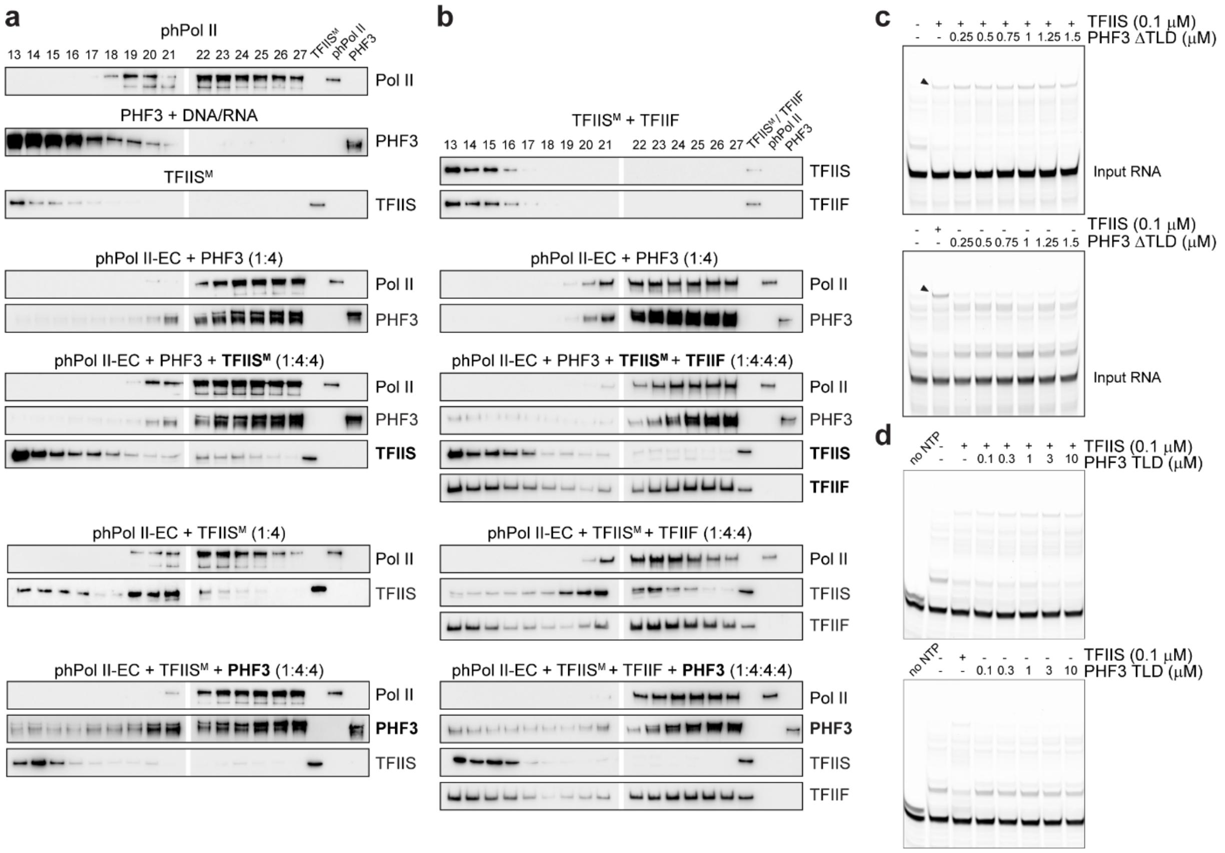

Supplementary Fig. 15. PHF3 competes with TFIIS. a,b, Competition between PHF3 and TFIIS for complex formation with phosphorylated Pol II-EC (elongation complex). Pol II was phosphorylated with DYRK1A and Pol II-EC was formed using a DNA-RNA bubble scaffold. Complexes with Pol II-EC were preformed by adding 4-fold molar excess of PHF3 or **a,** TFIIS^{M}, **b,** TFIIS^{M}+TFIIF. A 4-fold molar excess of the competitor indicated in bold was added to the preformed complexes. Complexes were separated by sucrose gradient ultracentrifugation and fractions were analysed by Western blotting. **c,d,** *In vitro* assay monitoring Pol II elongation on an arrest sequence in the presence or absence of TFIIS and increasing amounts of **c,** PHF3 ΔTLD, **d**, PHF3 TLD. Pol II-EC was formed using an excess of a DNA-RNA bubble scaffold containing 5'-FAM-labelled RNA. The short elongation product seen in the 'no NTP' lane is due to residual ATP from the phosphorylation reaction. All experiments were repeated twice with comparable results.

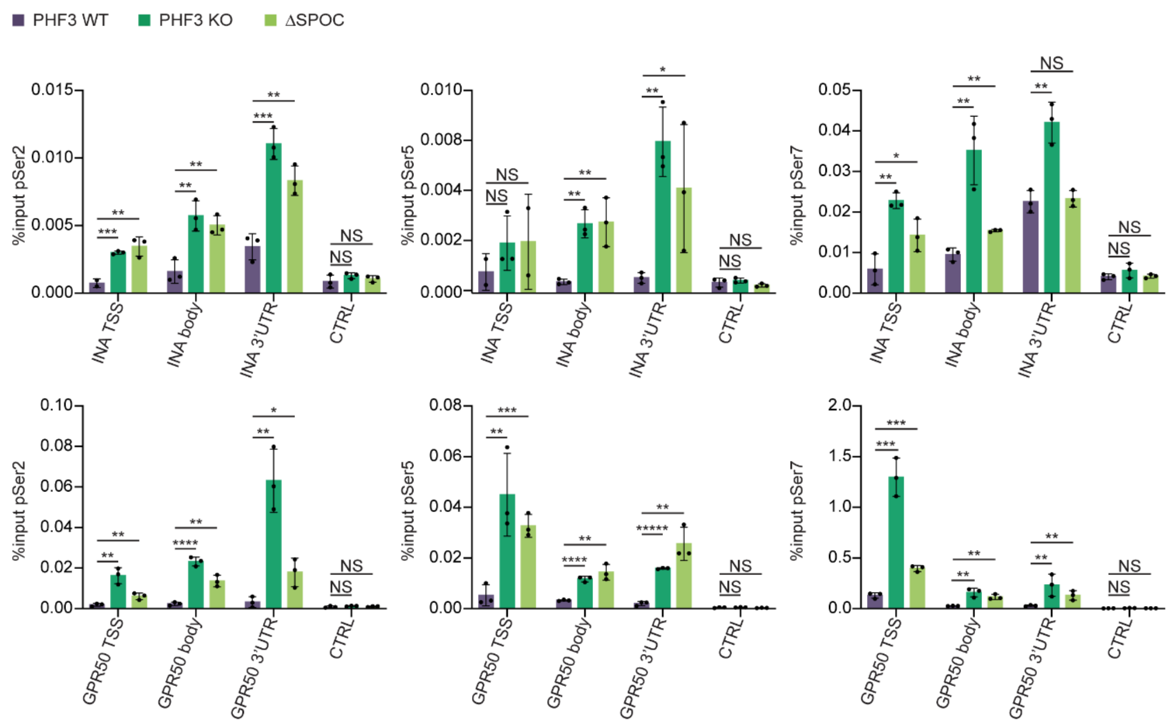

Supplementary Fig. 16. Pol II ChIP qPCR analysis of highly upregulated genes in PHF3 KO. ChIP-qPCR analysis of Pol II pSer2, pSer5 and pSer7 binding to different regions of the *INA* and *GPR50* genes and a negative control region in PHF3 WT, KO and ΔSPOC cells (mean±sd, n=3). The bars represent average Pol II occupancy from three biological replicates. A one-tailed, two-sample equal variance t-test was used to compare occupancy in KO and ΔSPOC to WT, statistical significance is indicated by asterisks (NS=not significant, *=P<0.05, **=P<0.01, ***=P<0.001, ****=P<0.0001, *****=P<0.00001). P-values are indicated in Supplementary Data 7.

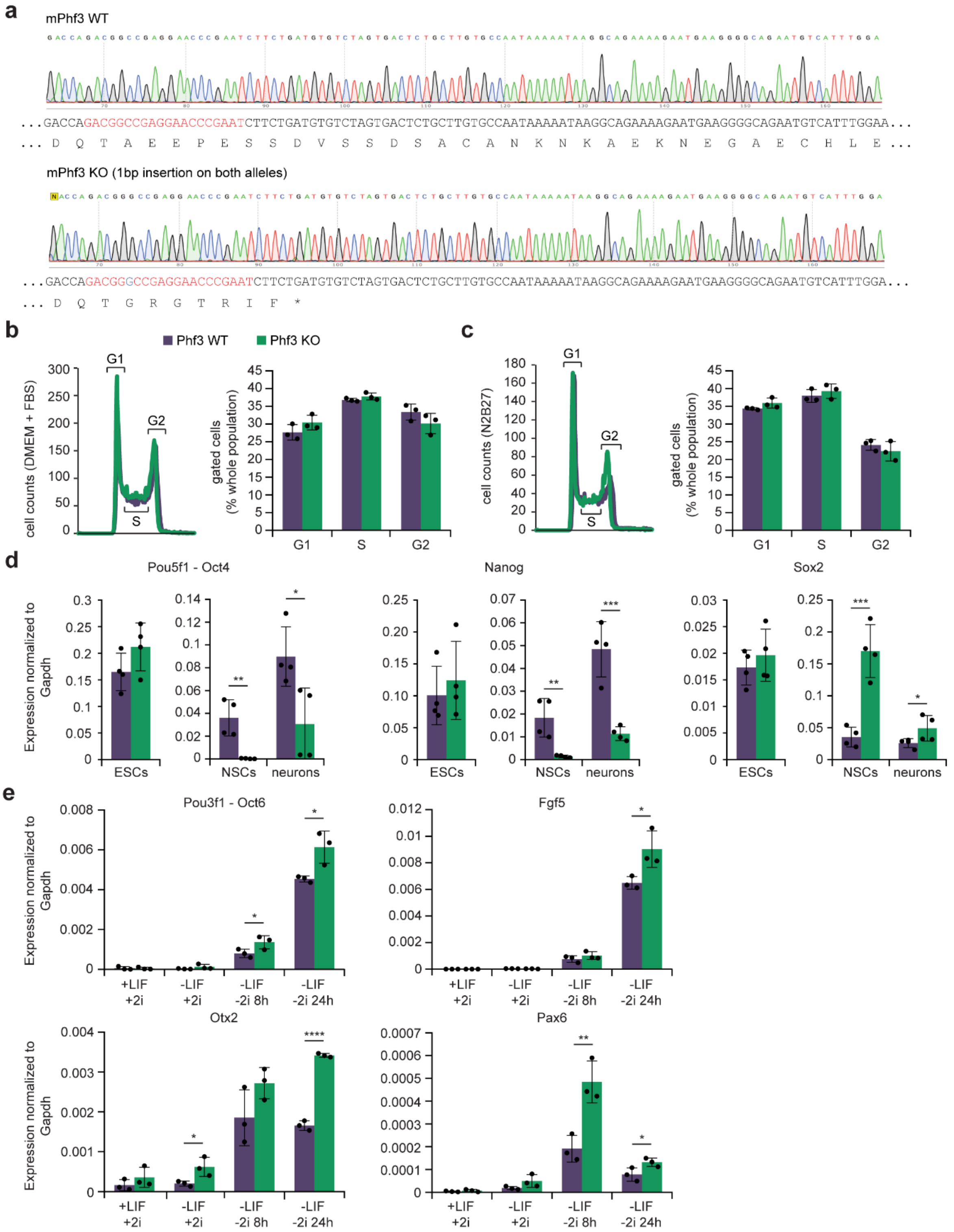

Supplementary Fig. 17. Generation of Phf3 KO mESCs and upregulation of early ectodermal markers in Phf3 KO ESCs. a, Sequencing snapshots and sequence reconstruction of mouse Phf3 KO in ESCs. **b,c** FACS analysis of cell cycle distribution of propidium iodide-stained Phf3 WT and KO mESCs grown in **b,** DMEM+FBS or **c,** N2B27 supplemented with 2i/LIF. The following cell numbers were examined over three independent experiments: N=24498 (WT _ DMEM + FBS), N=27008 (Phf3 KO _ DMEM + FBS), N=12693 (WT _ N2B27), N=13214 (Phf3 KO _ N2B27). **d,** RT-qPCR analysis of expression levels of stem cell markers Oct4, Nanog and Sox2 in Phf3 WT and KO during differentiation from mESCs to NSCs and neurons. Four biologically independent experiments were performed. Data are presented as mean values +/- standard deviation. **e,** RT-qPCR analysis of changes in expression levels of early ectodermal markers Oct6, Fgf5, Otx2 and Pax6 during 24 h differentiation of Phf3 WT and KO mESCs. Four biologically independent experiments were performed. Data are presented as mean values +/- standard deviation. One-tailed, two-sample equal variance t-test was used to determine p-values in **b, c, d, e**. P-values are indicated in Supplementary Data 7.

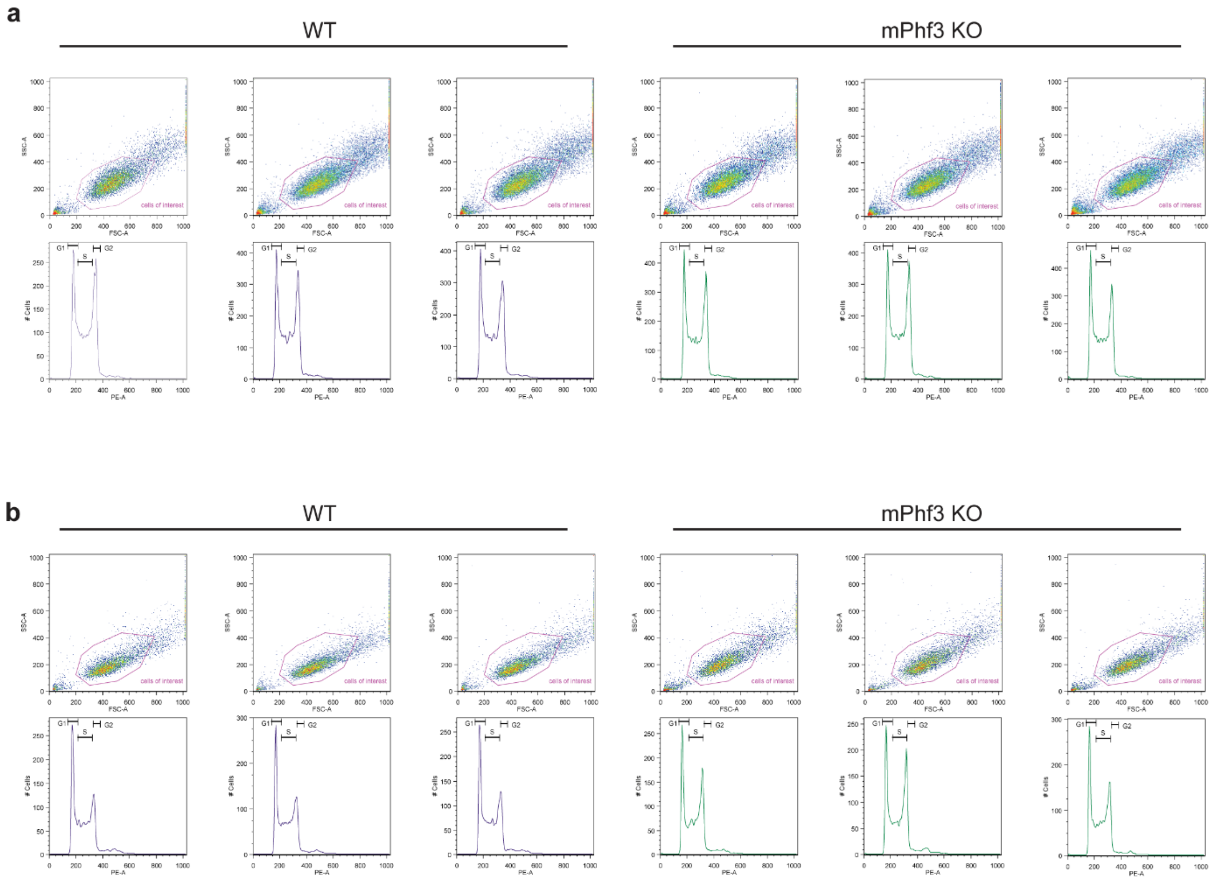

Supplementary Fig. 18. Gating strategy for the graphs in Supplementary Fig. 17b,c. a, Triplicates for WT (left) and mPhf3 KO (right) mESCs grown in DMEM+FBS+Lif medium are shown. The whole cell population is shown in the top row, with the gated population of interest. The gated cells of interest were then analyzed on the basis of the PE signal to quantify propidium iodide and quantify the cell percentage in different stages of the cell cycle (G1, S, G2 gates). **b,** Same as in a, for cells grown in N2B27+2i+Lif medium.

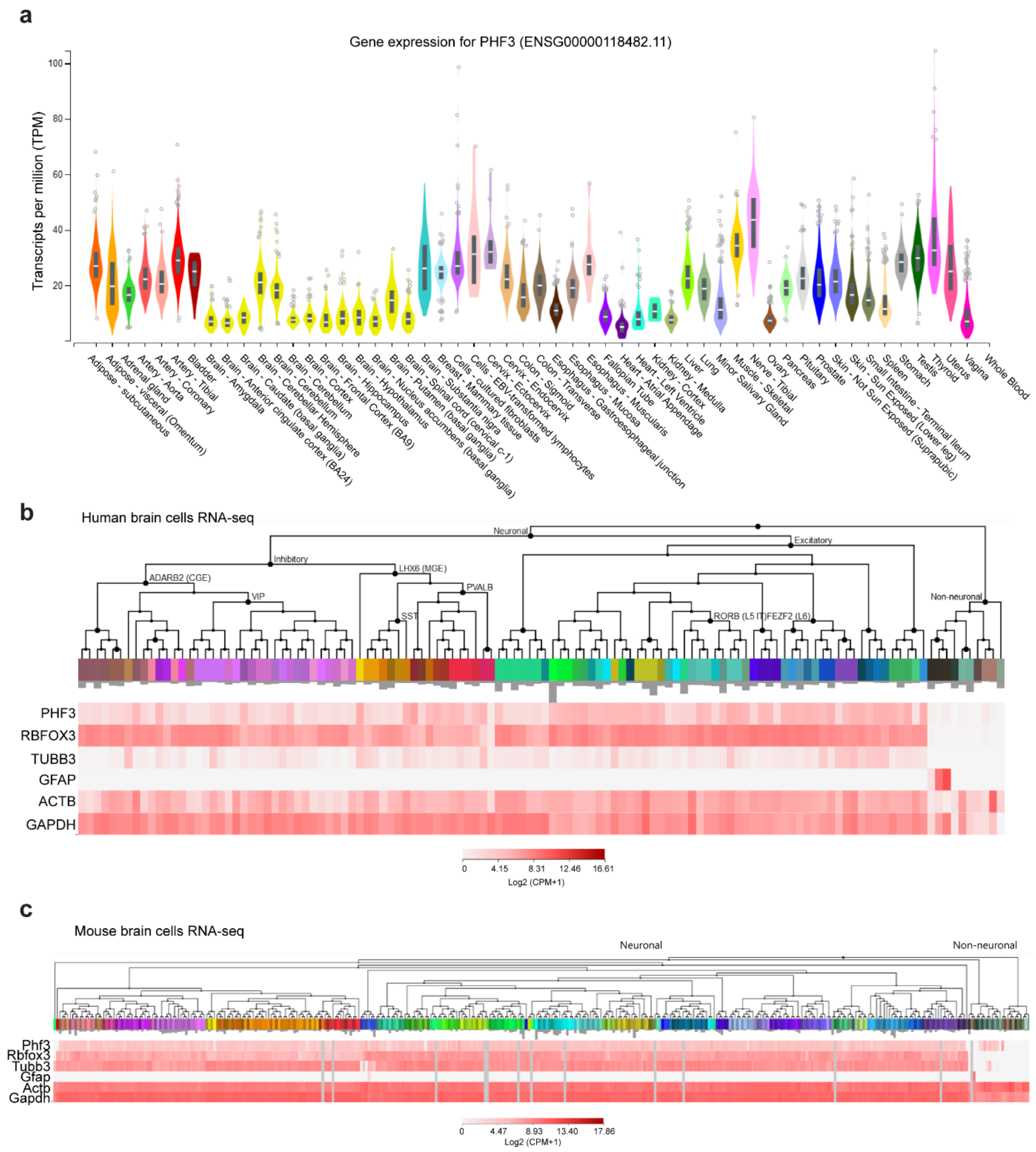

Supplementary Fig. 19. PHF3 expression levels across different tissues and in the brain. a, PHF3 gene expression levels across tissues based on GTEx Analysis Release V8. PHF3 shows on average a lower median expression in neural tissues. **b,c,** PHF3 expression in **b,** human and **c,** mouse brain cells from the Allen brain cell atlas. The heatmap shows the mean expression of PHF3 overlaid over human neuronal and non-neuronal cell types. Two pan neuronal markers, TUBB3 and RBFOX3, the astrocyte marker GFAP, and two housekeeping genes, ACTB and GAPDH, were also included.

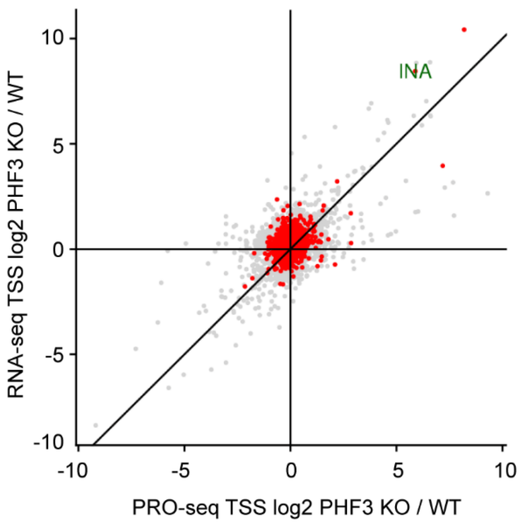

Supplementary Fig. 20. REST binding sites are not enriched on genes upregulated in PHF3 KO. Relationship between RNA-seq TSS fold change and PRO-seq TSS fold change for PHF3 KO vs WT. Genes with REST binding sites are indicated in red. Very few upregulated genes in PHF3 KO have REST binding sites, such as *INA* (indicated in green).

Supplementary References

1. Ariyoshi, M. & Schwabe, J.W. A conserved structural motif reveals the essential transcriptional repression function of Spen proteins and their role in developmental signaling. *Genes & development* **17**, 1909-1920 (2003).
