# Supplementary material for: PHF3 regulates neuronal gene expression through the Pol II CTD reader domain SPOC
Source: Nat Commun. 2021 Oct 19;12:6078. doi: 10.1038/s41467-021-26360-2 (PMC8526623; doi:10.1038/s41467-021-26360-2)
Supplement: Supplementary file 3 — Description of additional Supplementary Files [file 41467_2021_26360_MOESM3_ESM.pdf]

Description of Additional Supplementary Files

Supplementary Dataset 1:
Mass spectrometry analysis of PHF3 interactome in HEK293T cells.

Supplementary Dataset 2:
ChIP-seq of PHF3-GFP, Pol II F-12, pS2, pS5, pS7, TFIIS and H3K27me3 in PHF3 WT, KO and ΔSPOC HEK293T cells.

Supplementary Dataset 3:
PRO-seq and RNA-seq analysis in PHF3 WT, KO and ΔSPOC HEK293T cells.

Supplementary Dataset 4:
PRO-seq analysis of elongation rate in PHF3 WT, KO and ΔSPOC HEK293T cells.

Supplementary Dataset 5:
SLAM-seq analysis in PHF3 WT, KO and ΔSPOC HEK293T cells.

Supplementary Dataset 6:
RNA-seq analysis in mPHF3 WT and KO mES cells.

Supplementary Dataset 7:
Raw data for graphs, plots, gels and western blots shown in main and supplementary figures.
