# Supplementary material for: PHF3 regulates neuronal gene expression through the Pol II CTD reader domain SPOC
Source: Nat Commun. 2021 Oct 19;12:6078. doi: 10.1038/s41467-021-26360-2 (PMC8526623; doi:10.1038/s41467-021-26360-2)
Supplement: Supplementary file 11 — Reporting Summary [file 41467_2021_26360_MOESM11_ESM.pdf]

nature research

Corresponding author(s): Dea Slade

Last updated by author(s): Sep 17, 2021

Reporting Summary

Nature Research wishes to improve the reproducibility of the work that we publish. This form provides structure for consistency and transparency in reporting. For further information on Nature Research policies, see our Editorial Policies and the Editorial Policy Checklist.

Statistics

For all statistical analyses, confirm that the following items are present in the figure legend, table legend, main text, or Methods section.

n/a	Confirmed	
☐	☒	The exact sample size (*n*) for each experimental group/condition, given as a discrete number and unit of measurement
☐	☒	A statement on whether measurements were taken from distinct samples or whether the same sample was measured repeatedly
☐	☒	The statistical test(s) used AND whether they are one- or two-sided<br>*Only common tests should be described solely by name; describe more complex techniques in the Methods section.*
☒	☐	A description of all covariates tested
☐	☒	A description of any assumptions or corrections, such as tests of normality and adjustment for multiple comparisons
☐	☒	A full description of the statistical parameters including central tendency (e.g. means) or other basic estimates (e.g. regression coefficient) AND variation (e.g. standard deviation) or associated estimates of uncertainty (e.g. confidence intervals)
☐	☒	For null hypothesis testing, the test statistic (e.g. *F*, *t*, *r*) with confidence intervals, effect sizes, degrees of freedom and *P* value noted<br>*Give P values as exact values whenever suitable.*
☒	☐	For Bayesian analysis, information on the choice of priors and Markov chain Monte Carlo settings
☒	☐	For hierarchical and complex designs, identification of the appropriate level for tests and full reporting of outcomes
☐	☒	Estimates of effect sizes (e.g. Cohen's *d*, Pearson's *r*), indicating how they were calculated

Our web collection on statistics for biologists contains articles on many of the points above.

Software and code

Policy information about availability of computer code

Data collection

Western blots were imaged using ChemiDoc (BioRad). Mass spectrometry data were collected on Q Exactive (Thermo Fisher). Fluorescence anisotropy measurements were recorded using FluorEssence software (version 2.5.3.0 and V3.5, Horiba Jobin-Yvon). Mass photometry measurements were performed on a Refeyn OneMP mass photometer using AcquireMP software (v2.3.0). Condensates were imaged on Zeiss Axio Observer Z1 with a 60x oil immersion objective. Microscopy images were taken using Zeiss ZEN Black 3.2. FRAP analysis was performed on Zeiss AxioObserverZ1 equipped with a Yokogawa CSU-X1-A1 Nipkow spinning disc unit (pinhole diameter 50 µm, spacing 253 µm) (Visitron) with an EM-CCD: back-illuminated evolve EM512 highspeed/high-resolution camera (Evolve™ EMCCD; Photometrics) using an EC Plan-NeoFluor 100x/1.30NA Oil objective lens. Equipment control and imaging was handled by Visiview software (version 5.0.0.11; Visitron). Airyscan imaging was perfomed on an inverted Zeiss LSM 980 confocal microscope equipped with a 63x/1.4 Oil DIC objective and a 32 channel GaAsp Airyscan 2 detector unit controlled by Zen Black (software version 3.2). BD FACS Diva software 9.0 was used for FACS data collection. BioRad CFX384 Touch Real-Time PCR Detection System was used to acquire Real-Time qPCR data.

Data analysis

Western blots were analysed in Image Lab 6.0.1 (BioRad). Mass spectrometry data were analysed with Proteome Discoverer (version 1.4.0.288, Thermo Scientific), Mascot 2.2.07 (Matrix Science), SAINT-MS1 (doi: 10.1021/pr201185r) and Microsoft Excel 2016. Proteomic data were visualized with GeneMANIA. Fluorescence anisotropy data were analysed using GraphPad Prism 7 (GraphPad Software, La Jolla). FCS ACCESS Fit 1.0.12 (Carl Zeiss-Evotec) software package was used for FCS data analysis. Mass photometry data were processed and analysed using DiscoverMP software (v2.4.0). For X-ray crystallography, the data frames were processed using the XDS package version June 1, 2017 (doi: 10.1107/S0907444909047337) and converted to mtz format with the program AIMLESS (doi: 10.1107/S0907444910045749). The apo-SPOC structure was solved using single anomalous diffraction with the CRANK 2 software suite (doi: 10.1107/S0907444910052224). The structures of SPOC in complex with pS2, pS2pS5 and pS2pS7 CTD peptides were solved using the molecular replacement program PHASER (doi: 10.1107/S0021889807021206). The structures were refined with REFMAC (doi: 10.1107/S0907444996012255) and Phenix Refine version dev-3150 (doi: 10.1107/S0907444909052925) and rebuilt using Coot 0.8.9.1 (doi: 10.1107/S0907444904019158). CRANK2, AIMLESS, PHASER, REFMAC were distributed within the ccp4-7.0 version. The structures were validated and corrected using PDB_REDO server (doi: 10.1107/S2052252514009324). The figures were produced using the PyMol software 1.3 (Schrödinger). Microscopy images were processed with ImageJ 1.51. FRAP data was analysed using GraphPad Prism 6.04. Flowing Software version 2.5.1 was used for FACS data analysis. ChIP-

seq data were mapped to the hg38 version of the human genome using Bowtie2 as implemented in PigX pipeline v0.0.20 (doi: 10.1038/nmeth.1923). PRO-seq data were mapped to the hg38 version of the human reference genome using the STAR- 2.4.0 aligner. NET-seq data bigWig files were downloaded from the GEO database (GSE61332), and transferred from the hg19 to hg38 genome versions using CrossMap v0.3.8. RNA-seq data for HEK293T were mapped to the hg38 version of the human reference genome using STAR- 2.5.3. RNA-seq data for mESC were mapped to the mm9 version of the mouse reference genome using STAR- 2.4.0. RNA-seq data were quantified using STAR quantMode. Differential analysis was performed using DESeq2 1.24.0. SLAM-seq data was analysed using a SLAMdunk pipeline 0.3.4 (doi: 10.1038/nmeth.4435) and the RLM function from the MASS R package 7.3-51.4 (https://www.rdocumentation.org/packages/LVSmiRNA/versions/1.22.0/topics/RLM). The complete data integration and data analysis were done in R using Bioconductor1.30.4 (doi: 10.1038/nmeth.3252), and the following libraries: GenomicAlignments 1.20.1 (doi: 10.1371/journal.pcbi.1003118), data.table (Matt Dowle and Arun Srinivasan, 2017), data.table: Extension of `data.frame` (R package version 1.10.4-3.), biomaRt 2.40.4 (doi: 10.1038/nprot.2009.97), GenomicRanges 1.36.0, rtracklayer 1.44.0 (doi: 10.1093/bioinformatics/btp328), SummarizedExperiment 1.14.1 (10.18129/B9.bioc.SummarizedExperiment), genomation 1.16.0 (doi: 10.1093/bioinformatics/btu775), and ggplot2 3.2.1 (10.1007/978-0-387-98141-3). IGV browser shots were made with IGV version 2.4.1. Gene ontology analysis was performed with GSEA 3.0, MSigDB 6.1 'Biological processes' tool. BioRad CFX Maestro software 4.0.2325.0418 was used to determine the Ct for Real-Time qPCR experiments; data was exported and further analyzed in Microsoft Excel.

For manuscripts utilizing custom algorithms or software that are central to the research but not yet described in published literature, software must be made available to editors and reviewers. We strongly encourage code deposition in a community repository (e.g. GitHub). See the Nature Research guidelines for submitting code & software for further information.

Data

Policy information about availability of data

All manuscripts must include a data availability statement. This statement should provide the following information, where applicable:

- Accession codes, unique identifiers, or web links for publicly available datasets
- A list of figures that have associated raw data
- A description of any restrictions on data availability

The atomic coordinates have been deposited in the Protein Data Bank under accession codes: 6IC8 for PHF3 SPOC:2xpS2 [https://www.rcsb.org/structure/6IC8], 6IC9 for PHF3 SPOC:2xpS2pS7 [https://www.rcsb.org/structure/6IC9], 6Q2V for PHF3 SPOC [https://www.rcsb.org/structure/6Q2V], 6Q5Y for PHF3 SPOC:2xpS2pS5 [https://www.rcsb.org/structure/6Q5Y]. The sequencing data generated in this study have been deposited in ArrayExpress under accession codes: E-MTAB-7498 (RNA-seq HEK293T) [https://www.ebi.ac.uk/arrayexpress/experiments/E-MTAB-7498/], E-MTAB-8783 (PHF3) [https://www.ebi.ac.uk/arrayexpress/experiments/E-MTAB-8783/], E-MTAB-8789 (Pol II F-12, TFIIS, H3K27me3) [https://www.ebi.ac.uk/arrayexpress/experiments/E-MTAB-8789/], E-MTAB-7501 (PRO-seq) [https://www.ebi.ac.uk/arrayexpress/experiments/E-MTAB-7501/], E-MTAB-8278 (Pro-seq elongation rate) [https://www.ebi.ac.uk/arrayexpress/experiments/E-MTAB-8278/], E-MTAB-7898 [https://www.ebi.ac.uk/arrayexpress/experiments/E-MTAB-7898/] and E-MTAB-7899 (SLAM-seq) [https://www.ebi.ac.uk/arrayexpress/experiments/E-MTAB-7899/], E-MTAB-7526 (RNA-seq mESC) [https://www.ebi.ac.uk/arrayexpress/experiments/E-MTAB-7526/]. The mass spectrometry proteomics data generated in this study have been deposited in the ProteomeXchange Consortium via the PRIDE partner repository under accession code PXD026292. The processed mass spectrometry and sequencing data are provided in Supplementary Data 1-6. All the raw data generated in this study are provided in Supplementary Data 7. Atomic coordinates used in this study are available in the Protein Data Bank under accession codes 2RT5 [https://www.rcsb.org/structure/2RT5], 4BY7 [https://www.rcsb.org/structure/4BY7], 5KXF [https://www.rcsb.org/structure/5KXF], 5IYB [https://www.rcsb.org/structure/5IYB], 6GMH [https://www.rcsb.org/structure/6GMH], [6IC8 https://www.rcsb.org/structure/6IC8]. The NET-seq data used in this study are available in GEO under accession code GSE61332 [https://www.ncbi.nlm.nih.gov/geo/query/acc.cgi?acc=GSE61332]. The ATAC-seq data used in this study are available in ArrayExpress under accession code E-MTAB-6195 [https://www.ebi.ac.uk/arrayexpress/experiments/E-MTAB-6195/]. H3K4me3 ChIP-seq data used in this study are available from ENCODE under accession code ENCSR000DTU [https://www.encodeproject.org/experiments/ENCSR000DTU/]. REST ChIP-seq data used in this study are available from ENCODE under accession code ENCSR896UBV [https://www.encodeproject.org/search/?searchTerm=ENCSR896UBV].

Field-specific reporting

Please select the one below that is the best fit for your research. If you are not sure, read the appropriate sections before making your selection.

☒ Life sciences ☐ Behavioural & social sciences ☐ Ecological, evolutionary & environmental sciences

For a reference copy of the document with all sections, see nature.com/documents/nr-reporting-summary-flat.pdf

Life sciences study design

All studies must disclose on these points even when the disclosure is negative.

Sample size	No statistical methods were used to predetermine sample size. All experiments have been performed minimally as triplicates, because triplicate experimental design allows for outlier detection (detection of failed experiments). Triplicate experiments per biological condition are sufficient for ascertaining statistical significance with the fold change of interest (e.g., 2-fold change).
Data exclusions	No data were excluded from the analyses.
Replication	All attempts at replication were successful. All experiments were replicated three to six times.
Randomization	Randomization was not used, as it is not applicable to the experiments performed in this study. Randomization is required for in vivo experiments, while all experiments in this manuscript were performed in vitro.
Blinding	The investigators were not blinded during data acquisition or analysis as this is not required for the experiments performed in this study. Analyses were done in an automated fashion eliminating human bias.

Reporting for specific materials, systems and methods

We require information from authors about some types of materials, experimental systems and methods used in many studies. Here, indicate whether each material, system or method listed is relevant to your study. If you are not sure if a list item applies to your research, read the appropriate section before selecting a response.

Materials & experimental systems

n/a	Involved in the study
☐	☒ Antibodies
☐	☒ Eukaryotic cell lines
☒	☐ Palaeontology and archaeology
☒	☐ Animals and other organisms
☒	☐ Human research participants
☒	☐ Clinical data
☒	☐ Dual use research of concern

Methods

n/a	Involved in the study
☐	☒ ChIP-seq
☐	☒ Flow cytometry
☒	☐ MRI-based neuroimaging

Antibodies

Antibodies used

For Western blotting: Anti-FLAG M2-peroxidase clone M2 (1:10000; Sigma A8592), rabbit anti-PHF3 (1:500; Sigma HPA025763), rabbit anti-pS2 Pol II (1:1000; Bethyl laboratories A300-654A), rat anti-pS2 Pol II clone 3E10 (1:1000; Millipore 04-1571), mouse anti-pS5 Pol II clone 4H8 (1:1000; Cell Signaling 2629), rat anti-pS7 Pol II clone 4E12 (1:1000; Millipore 04-1570), rat anti-pS5 Pol II clone 3E8 (1:1000; Millipore 04-1572), mouse anti-Pol II clone F-12 (1:1000; Santa Cruz sc-55492), mouse 8WG16 (1:1000; ab817), rabbit anti-TFIIF (1:1000; ab28179), mouse anti-TCEA1 (1:1000; Santa Cruz sc-393439), mouse anti-α-tubulin clone B512 (1:5000; Sigma T6074), mouse anti-GFP clone 2B6 (1:1000; Egon Ogris lab) and rabbit anti-GFP (1:1000; ab290), rabbit anti-SPT6 (1:1000; Novus NB100-2582), mouse anti-DSIF (1:1000; BD 611107), rabbit anti-PAF1 (1:1000; ab20662), rabbit anti-SPT16 (1:1000; Santa Cruz sc-28734), mouse anti-SSRP1 (1:1000; ab26212).

For immunofluorescence: mouse anti-FLAG M2 (1:500; Sigma F1804), rabbit anti-PHF3 (1:100; Sigma HPA025763), rabbit anti-β-Tubulin III TuJ1 (1:500; Sigma T2200), mouse anti-GFAP (1:500; Sigma G3893), rat anti-pS2 Pol II clone 3E10 (1:500; Millipore 04-1571), rat anti-pS5 Pol II clone 3E8 (1:1000; Millipore 04-1572), rabbit anti-GFP (1:500; ab290), mouse anti-Pol II clone F-12 (1:200; Santa Cruz sc-55492), rabbit anti-H3K27me3 (1:500; Millipore 07-449).

Secondary HRP-conjugated antibodies for Western blotting (Jackson ImmunoResearch goat anti-rabbit 111-035-008, goat anti-mouse 115-035-008, goat anti-rat 112-035-008) were used at 1:10000 dilution.

Secondary Alexa Fluor® antibodies for immunofluorescence (Invitrogen goat anti-mouse Alexa Fluor 488 A11001, goat anti-rabbit Alexa Fluor 488 A11008, goat anti-mouse Alexa Fluor 568 A11004, goat anti-rabbit Alexa Fluor 568 A11011, goat anti-rat Alexa Fluor 594 A11007) were used at 1:500 dilution.

Validation

Antibodies were validated by the manufacturer and used extensively in the literature or validated in this manuscript.

Mouse anti-FLAG M2 (Sigma) - Manufacturer's statement on Specificity: 'Detects a single band of protein on a western blot from an E. coli crude cell lysate.'

Rabbit anti-PHF3 (Atlas Antibodies) - validated in this manuscript by showing no signal in PHF3 KO (WB and IF) and specific recognition of in vitro purified PHF3 (WB)

Rat phosphoCTD 3E8, 3E10, 4E12 were validated in DOI: 10.1126/science.1145977

Mouse anti-Pol II 4H8 (Cell Signaling) - Manufacturer's statement on Specificity: 'Rpb1 CTD (4H8) Antibody detects endogenous levels of total Rpb1 protein (both phosphorylated and unphosphorylated forms).'

Mouse anti-Pol II F-12 (Santa Cruz) - used in >30 publications and validated in this manuscript using purified Pol II complex in WB and in ChIP

Mouse anti-Pol II 8WG16 (abcam) - validated in this manuscript using purified Pol II complex (WB)

Rabbit anti-TFIIF (abcam) - validated in this manuscript using purified TFIIF (WB)

Rabbit anti-H3K27me3 (Millipore) - validated by the manufacturer: 'Protein A purified antibody is dot blot tested for trimethylated lysine 27 specificity and validated in WB, ICC, IP.'

Mouse anti-TCEA1 (Santa Cruz) - validated in this manuscript using purified TFIIS (WB)

Mouse anti-TCEA1 (abcam) - validated in this manuscript by ChIP

Mouse anti-α-tubulin clone B512 (Sigma) - used in >100 publications

Mouse anti-GFP 2B6 (Egon Ogris) - Evaluated by Western Blotting in lysates from HeLa cells transfected with GFP. Western Blotting Analysis: 1 µg/mL of this antibody detected GFP in lysates from HeLa cells transfected with GFP.

Mouse anti-GFP (abcam) - Manufacturer's statement on Specificity: 'On Western blot the antibody detects the GFP fraction from cell extracts expressing recombinant GFP fusion proteins and has also been shown to be useful on mouse sections fixed with formalin. In Immunocytochemistry, the antibody gives a very good signal on recombinant YES-GFP chimeras expressed in COS cells (McCabe et al. 1999). It is routinely used in Immunoprecipitation (IP) and IP-Western protocols and has been used successfully in HRP Immunohistochemistry at 1:200 on whole-mount mouse embryos. This antibody is reactive against all variants of Aequorea victoria GFP such as S65T-GFP, RS-GFP and EGFP.'

Rabbit anti-SPT6 (Novus) - Validated by the manufacturer using siRNA knockdown to confirm the specificity of Spt6 antibody in C2C12 myoblasts

Mouse anti-DSIF (BD) - Manufacturer's statement: 'This antibody is routinely tested by western blot analysis.'

Rabbit anti-PAF1 (abcam) - Manufacturer's statement (WB): 'ab20662 recognizes a band at approximately 80kDa, which corresponds in size to PAF1 / PD2. Although it has a calculated molecular weight of ~60kDa, several groups have shown that it migrates at a size of 80-90kDa (see Moniaux, et.al., Oncogene Feb. 2006, Yart et.al., Mol Cell Biol 25:5052-60, 2005, and Rozenblatt-Rosen, et.al., Mol Cell

Biol 25:612-20, 2005). There is also a smaller band at ~73kDa which may be a degradation product. Both bands are competed away by the addition of immunizing peptide, suggesting that the interaction is specific.'
Rabbit anti-β-Tubulin III TuJ1 (Sigma) - knockout validation by the manufacturer
Mouse anti-GFAP (Sigma) - Manufacturer's statement on Specificity: 'The antibody reacts specifically with GFAP in immunoblotting assays and labels astrocytes, Bergmann glia cells and chondrocytes of elastic cartilage in immunohistochemical staining. The antibody reacts with glial specific antigen in frozen or alcohol-fixed tissue sections.'

Eukaryotic cell lines

Policy information about cell lines

Cell line source(s)	HEK293T were obtained from ATCC. Rex1GFPd2::Cas9 (RC9) ES cells were used that carry a destabilized GFP-reporter for Rex1-expression and a stably expressed Cas9 transgene integrated into the Rosa26 locus (doi: 10.1038/ncb2267; doi: 10.1016/j.celrep.2018.06.027). SF9 (94-001F) were from Expression Systems.
Authentication	mESCs were authenticated by measuring the expression of known pluripotency marker genes by qPCR. HEK293T and SF9 were not authenticated.
Mycoplasma contamination	The cell lines tested negative for Mycoplasma contamination based on MycoAlert Mycoplasma detection kit (Lonza), DAPI staining and PCR.
Commonly misidentified lines (See ICLAC register)	No commonly misidentified cell lines were used in this study.

ChIP-seq

Data deposition

- [x] Confirm that both raw and final processed data have been deposited in a public database such as GEO.
- [x] Confirm that you have deposited or provided access to graph files (e.g. BED files) for the called peaks.

Data access links
May remain private before publication.

http://www.ebi.ac.uk/arrayexpress/experiments/E-MTAB-8783
Username: Reviewer_E-MTAB-8783
Password: xkdthhga

http://www.ebi.ac.uk/arrayexpress/experiments/E-MTAB-8789
Username: Reviewer_E-MTAB-8789
Password: trhreba1

Files in database submission

ChIP_HEK_KO_input_br1.fastq.gz
ChIP_HEK_KO_input_br2.fastq.gz
ChIP_HEK_KO_input_br3.fastq.gz
ChIP_HEK_KO_pSer2_br1.fastq.gz
ChIP_HEK_KO_pSer2_br2.fastq.gz
ChIP_HEK_KO_pSer2_br3.fastq.gz
ChIP_HEK_KO_pSer5_br1.fastq.gz
ChIP_HEK_KO_pSer5_br2.fastq.gz
ChIP_HEK_KO_pSer5_br3.fastq.gz
ChIP_HEK_KO_pSer7_br1.fastq.gz
ChIP_HEK_KO_pSer7_br2.fastq.gz
ChIP_HEK_KO_pSer7_br3.fastq.gz
ChIP_HEK_SPOC_input_br1.fastq.gz
ChIP_HEK_SPOC_input_br2.fastq.gz
ChIP_HEK_SPOC_input_br3.fastq.gz
ChIP_HEK_SPOC_pSer2_br1.fastq.gz
ChIP_HEK_SPOC_pSer2_br2.fastq.gz
ChIP_HEK_SPOC_pSer2_br3.fastq.gz
ChIP_HEK_SPOC_pSer5_br1.fastq.gz
ChIP_HEK_SPOC_pSer5_br2.fastq.gz
ChIP_HEK_SPOC_pSer5_br3.fastq.gz
ChIP_HEK_SPOC_pSer7_br1.fastq.gz
ChIP_HEK_SPOC_pSer7_br2.fastq.gz
ChIP_HEK_SPOC_pSer7_br3.fastq.gz
ChIP_HEK_WT_input_br1.fastq.gz
ChIP_HEK_WT_input_br2.fastq.gz

ChIP_HEK_WT_input_br3.fastq.gz
ChIP_HEK_WT_PHF3_br1.fastq.gz
ChIP_HEK_WT_PHF3_br2.fastq.gz
ChIP_HEK_WT_PHF3_br3.fastq.gz
ChIP_HEK_WT_PHF3.GFP_br1.fastq.gz
ChIP_HEK_WT_PHF3.GFP_br2.fastq.gz
ChIP_HEK_WT_PHF3.GFP_br3.fastq.gz
ChIP_HEK_WT_pSer2_br1.fastq.gz
ChIP_HEK_WT_pSer2_br2.fastq.gz
ChIP_HEK_WT_pSer2_br3.fastq.gz
ChIP_HEK_WT_pSer5_br1.fastq.gz
ChIP_HEK_WT_pSer5_br2.fastq.gz
ChIP_HEK_WT_pSer5_br3.fastq.gz
ChIP_HEK_WT_pSer7_br1.fastq.gz
ChIP_HEK_WT_pSer7_br2.fastq.gz
ChIP_HEK_WT_pSer7_br3.fastq.gz
ChIP_HEK_KO_F12_br1.fastq.gz
ChIP_HEK_KO_F12_br2.fastq.gz
ChIP_HEK_KO_F12_br3.fastq.gz
ChIP_HEK_KO_H3K27me3_br1.fastq.gz
ChIP_HEK_KO_H3K27me3_br2.fastq.gz
ChIP_HEK_KO_H3K27me3_br3.fastq.gz
ChIP_HEK_KO_TFIIS_br1.fastq.gz
ChIP_HEK_KO_TFIIS_br2.fastq.gz
ChIP_HEK_KO_TFIIS_br3.fastq.gz
ChIP_HEK_SPOC_F12_br1.fastq.gz
ChIP_HEK_SPOC_F12_br2.fastq.gz
ChIP_HEK_SPOC_F12_br3.fastq.gz
ChIP_HEK_SPOC_H3K27me3_br1.fastq.gz
ChIP_HEK_SPOC_H3K27me3_br2.fastq.gz
ChIP_HEK_SPOC_H3K27me3_br3.fastq.gz
ChIP_HEK_SPOC_TFIIS_br1.fastq.gz
ChIP_HEK_SPOC_TFIIS_br2.fastq.gz
ChIP_HEK_SPOC_TFIIS_br3.fastq.gz
ChIP_HEK_WT_F12_br1.fastq.gz
ChIP_HEK_WT_F12_br2.fastq.gz
ChIP_HEK_WT_F12_br3.fastq.gz
ChIP_HEK_WT_H3K27me3_br1.fastq.gz
ChIP_HEK_WT_H3K27me3_br2.fastq.gz
ChIP_HEK_WT_H3K27me3_br3.fastq.gz
ChIP_HEK_WT_TFIIS_br1.fastq.gz
ChIP_HEK_WT_TFIIS_br2.fastq.gz
ChIP_HEK_WT_TFIIS_br3.fastq.gz

Genome browser session
(e.g. UCSC)

http://bimsbstatic.mdc-berlin.de/akalin/vfranke/DSla/UCSC_Hub/hub.txt

Methodology

Replicates

Every ChIP experiment was repeated three times.

Sequencing depth

ChIP_HEK_KO_input_br1.fastq.gz 98693211
ChIP_HEK_KO_input_br2.fastq.gz 78975438
ChIP_HEK_KO_input_br3.fastq.gz 71763476
ChIP_HEK_KO_pSer2_br1.fastq.gz 88830336
ChIP_HEK_KO_pSer2_br2.fastq.gz 68005778
ChIP_HEK_KO_pSer2_br3.fastq.gz 120742894
ChIP_HEK_KO_pSer5_br1.fastq.gz 138756669
ChIP_HEK_KO_pSer5_br2.fastq.gz 92270343
ChIP_HEK_KO_pSer5_br3.fastq.gz 50893403
ChIP_HEK_KO_pSer7_br1.fastq.gz 76500113
ChIP_HEK_KO_pSer7_br2.fastq.gz 37684345
ChIP_HEK_KO_pSer7_br3.fastq.gz 79759210
ChIP_HEK_SPOC_input_br1.fastq.gz 64916438
ChIP_HEK_SPOC_input_br2.fastq.gz 60512158
ChIP_HEK_SPOC_input_br3.fastq.gz 49318478
ChIP_HEK_SPOC_pSer2_br1.fastq.gz 64351575
ChIP_HEK_SPOC_pSer2_br2.fastq.gz 98071566
ChIP_HEK_SPOC_pSer2_br3.fastq.gz 36762463

ChIP_HEK_SPOC_pSer5_br1.fastq.gz 41726733
ChIP_HEK_SPOC_pSer5_br2.fastq.gz 38800532
ChIP_HEK_SPOC_pSer5_br3.fastq.gz 129376989
ChIP_HEK_SPOC_pSer7_br1.fastq.gz 113692371
ChIP_HEK_SPOC_pSer7_br2.fastq.gz 72801628
ChIP_HEK_SPOC_pSer7_br3.fastq.gz 58171917
ChIP_HEK_WT_input_br1.fastq.gz 58791462
ChIP_HEK_WT_input_br2.fastq.gz 54665065
ChIP_HEK_WT_input_br3.fastq.gz 62102968
ChIP_HEK_WT_PHF3_br1.fastq.gz 57096873
ChIP_HEK_WT_PHF3_br2.fastq.gz 56629853
ChIP_HEK_WT_PHF3_br3.fastq.gz 107872226
ChIP_HEK_WT_PHF3.GFP_br1.fastq.gz 56737845
ChIP_HEK_WT_PHF3.GFP_br2.fastq.gz 56727743
ChIP_HEK_WT_PHF3.GFP_br3.fastq.gz 105137385
ChIP_HEK_WT_pSer2_br1.fastq.gz 82242848
ChIP_HEK_WT_pSer2_br2.fastq.gz 70419230
ChIP_HEK_WT_pSer2_br3.fastq.gz 81654226
ChIP_HEK_WT_pSer5_br1.fastq.gz 71014932
ChIP_HEK_WT_pSer5_br2.fastq.gz 41335283
ChIP_HEK_WT_pSer5_br3.fastq.gz 77433075
ChIP_HEK_WT_pSer7_br1.fastq.gz 74516915
ChIP_HEK_WT_pSer7_br2.fastq.gz 82588738
ChIP_HEK_WT_pSer7_br3.fastq.gz 65911520
ChIP_HEK_KO_F12_br1.fastq.gz 37162488
ChIP_HEK_KO_F12_br2.fastq.gz 104803736
ChIP_HEK_KO_F12_br3.fastq.gz 53812071
ChIP_HEK_KO_H3K27me3_br1.fastq.gz 42478255
ChIP_HEK_KO_H3K27me3_br2.fastq.gz 59452564
ChIP_HEK_KO_H3K27me3_br3.fastq.gz 75319392
ChIP_HEK_KO_TFIIS_br1.fastq.gz 51473248
ChIP_HEK_KO_TFIIS_br2.fastq.gz 49801115
ChIP_HEK_KO_TFIIS_br3.fastq.gz 45876864
ChIP_HEK_SPOC_F12_br1.fastq.gz 36758255
ChIP_HEK_SPOC_F12_br2.fastq.gz 51436437
ChIP_HEK_SPOC_F12_br3.fastq.gz 52387158
ChIP_HEK_SPOC_H3K27me3_br1.fastq.gz 41866879
ChIP_HEK_SPOC_H3K27me3_br2.fastq.gz 43870747
ChIP_HEK_SPOC_H3K27me3_br3.fastq.gz 80844969
ChIP_HEK_SPOC_TFIIS_br1.fastq.gz 48600334
ChIP_HEK_SPOC_TFIIS_br2.fastq.gz 46494317
ChIP_HEK_SPOC_TFIIS_br3.fastq.gz 45231045
ChIP_HEK_WT_F12_br1.fastq.gz 39825150
ChIP_HEK_WT_F12_br2.fastq.gz 61454585
ChIP_HEK_WT_F12_br3.fastq.gz 62815966
ChIP_HEK_WT_H3K27me3_br1.fastq.gz 50916318
ChIP_HEK_WT_H3K27me3_br2.fastq.gz 39224745
ChIP_HEK_WT_H3K27me3_br3.fastq.gz 83942510
ChIP_HEK_WT_TFIIS_br1.fastq.gz 52725884
ChIP_HEK_WT_TFIIS_br2.fastq.gz 64189909
ChIP_HEK_WT_TFIIS_br3.fastq.gz 42812037

Antibodies

Pol II F-12 (Santa Cruz sc-55492)
pSer2 (3E10)
pSer5 (3E8)
pSer7 (4E12)
TFIIS (ab185947)
H3K27me3 (Millipore 07-449)
GFP (Abcam ab290)

Peak calling parameters

Peak calling software was not used for the analysis of ChIP-seq data. DESeq2 was used to estimate the enrichment in different genomic regions, measured as log2 ChIP / Input.

Data quality

Data quality assessment is provided in Extended Data Figure 8.

Software

ChIP-seq data were mapped to the hg38 version of the human genome using Bowtie2 (doi: 10.1038/nmeth.1923). R and Python scripts will be made available upon request.

Flow Cytometry

Plots

Confirm that:

☐ The axis labels state the marker and fluorochrome used (e.g. CD4-FITC).

☐ The axis scales are clearly visible. Include numbers along axes only for bottom left plot of group (a 'group' is an analysis of identical markers).

☐ All plots are contour plots with outliers or pseudocolor plots.

☒ A numerical value for number of cells or percentage (with statistics) is provided.

Methodology

Sample preparation	HEK293T PHF3 WT, KO and ΔSPOC cells were grown for 24 h in 24-well plates. Cells were incubated with 0.5 µM EU (Molecular Probes) for 1 h. Cells were harvested by trypsinization, washed in PBS and fixed overnight in 75% methanol at -20° C. Fixed cells were washed with PBS, blocked in 3% BSA and permeabilized in 0.25% Triton X-100 in PBS. Click-iT reaction was performed to couple Alexa Fluor 488 Azide to the incorporated EU. Cells were subsequently washed twice in 3% BSA in PBS and finally resuspended in PBS.<br>mESCs were grown in 24-well plates, harvested by trypsinization, washed with PBS and fixed overnight in 75% methanol at -20°C. Fixed cells were washed with PBS, blocked in 3% BSA and permeabilized in 0.25% Triton X-100 in PBS. Cells were resuspended in propidium iodide solution.
Instrument	BD LSRFortessa
Software	BD FACS Diva software was used for data collection, Flowing Software version 2.5.1 was used for data analysis.
Cell population abundance	From the main FSC and SSC gated population, events up to 10.000 were recorded and used for further analysis.
Gating strategy	Cells were evaluated according to the FSC and SSC parameters. The major homogeneous cell population was encircled according to FSC and SSC and used for data analysis. For EU experiment, the negative -EU control is provided, together with the P1 gating strategy.

☒ Tick this box to confirm that a figure exemplifying the gating strategy is provided in the Supplementary Information.
